# Recommendations for prediction models in clinical practice guidelines for cardiovascular diseases are over-optimistic: a global survey utilizing a systematic literature search

**DOI:** 10.3389/fcvm.2024.1449058

**Published:** 2024-10-17

**Authors:** Cheng-yang Jing, Le Zhang, Lin Feng, Jia-chen Li, Li-rong Liang, Jing Hu, Xing Liao

**Affiliations:** ^1^ Center for Evidence Based Chinese Medicine, Institute of Basic Research in Clinical Medicine, China Academy of Chinese Medical Sciences, Beijing, China; ^2^ Department of Clinical Epidemiology, Beijing Institute of Respiratory Medicine and Beijing Chao-Yang Hospital, Capital Medical University, Beijing, China; ^3^ Beijing Institute of Traditional Chinese Medicine, Beijing Hospital of Traditional Chinese Medicine, Capital Medical University, Beijing, China

**Keywords:** prediction model, recommendation, clinical practice guideline, cardiovascular diseases, methodological quality, risk of bias

## Abstract

**Background:**

This study aimed to synthesize the recommendations for prediction models in cardiovascular clinical practice guidelines (CPGs) and assess the methodological quality of the relevant primary modeling studies.

**Methods:**

We performed a systematic literature search of all available cardiovascular CPGs published between 2018 and 2023 that presented specific recommendations (whether in support or non-support) for at least one multivariable clinical prediction model. For the guideline-recommended models, the assessment of the methodological quality of their primary modeling studies was conducted using the Prediction model Risk Of Bias ASsessment Tool (PROBAST).

**Results:**

In total, 46 qualified cardiovascular CPGs were included, with 69 prediction models and 80 specific recommendations. Of the 80 specific recommendations, 74 supported 57 models (53 were fully recommended and 4 were conditionally recommended) in cardiovascular practice with moderate to strong strength. Most of the guideline-recommended models were focused on predicting prognosis outcomes (53/57, 93%) in primary and tertiary prevention, focusing primarily on long-term risk stratification and prognosis management. A total of 10 conditions and 7 types of target population were involved in the 57 models, while heart failure (14/57, 25%) and a general population with or without cardiovascular risk factor(s) (12/57, 21%) received the most attention from the guidelines. The assessment of the methodological quality of 57 primary studies on the development of the guideline-recommended models revealed that only 40% of the modeling studies had a low risk of bias (ROB). The causes of high ROB were mainly in the analysis and participant domains.

**Conclusions:**

Global cardiovascular CPGs presented an unduly positive appraisal of the existing prediction models in terms of ROB, leading to stronger recommendations than were warranted. Future cardiovascular practice may benefit from well-established clinical prediction models with better methodological quality and extensive external validation.

## 1 Introduction

Cardiovascular diseases (CVDs) are major causes of mortality and a leading contributor to disability globally with the burden of disease continuing its decades-long rise in almost all countries (1, 2). In the United States of America, the estimated cost due to CVD for healthcare services, medications, and lost productivity was over $200 billion annually (3). This growing burden of CVD can be broadly attributed to the suboptimal implementation of prevention strategies and poor management of cardiovascular risk factors (3, 4). Hence, the assessment of CVD risk in the general population, risk stratification in suspected patients, and prevention of recurrence in patients suffering from CVD represent an opportunity for major public health gains (1, 5).

Multivariable clinical prediction models (also known as risk prediction/assessment tools or prediction rules) are usually developed based on person-level information, aiming to estimate the pretest probability or risk of an individual having (diagnosis) or developing (prognosis) a particular outcome (6–8). As they play an increasingly vital role in the screening, diagnosis, and prognosis management of conditions, prediction models have gradually received increased attention from healthcare service providers and creators of clinical practice guidelines (CPGs), especially in the cardiovascular domain (9, 10). Beyond their clinical utility, their economic value has also been demonstrated in a considerable number of pharmacoeconomics analyses (11). Currently, quite a few available prediction models have been recommended for clinical practice in cardiovascular CPGs, e.g., there are 14 models recommended for the primary and tertiary prevention of heart failure (HF) in the 2022 American Heart Association (AHA)/American College of Cardiology (ACC)/Heart Failure Society of America (HFSA) Guideline for the Management of HF (12). Furthermore, other well-known models, e.g., the Framingham Risk Score (FRS), Systematic Coronary Risk Estimation 2 (SCORE2), Pooled Cohort Equations (PCE), and the QRISK3 tool, are widely recommended for the assessment of 10-year CVD risk in European and American populations with or without risk factor(s) (13–16).

Despite the promising application prospects of the prediction models, the methodological quality of the primary studies on the development of the models has been roundly criticized in recent years. In the different medical domains, almost all the primary modeling studies are at high risk of bias (ROB), whether the models were developed based on traditional regression methods (e.g., Logistic regression) or emerging artificial intelligence (AI) technology (e.g., machine learning algorithms) (17–20). The same is true without discrepancy in the cardiovascular domain (21–23). Given the enormous impact of CPGs on clinical practice (24), an assessment of the methodological quality of primary modeling studies related to guideline-recommended models appears to be necessary.

Prior reports mainly reviewed and summarized several prediction models for cardiovascular risk classification in primary prevention, with limitations in their application to other clinical settings and target populations/conditions (10, 15, 25). To date, the recommendations for prediction models in cardiovascular practice guidelines have not been adequately synthesized, nor has methodological quality assessment been systematically conducted for primary modeling studies. Thus, we performed a systematic literature search aiming to (1) identify and provide an overview of the recommendations for prediction models in cardiovascular CPGs, (2) summarize the characteristics of the involved models in clinical practice, and (3) assess the ROB of the primary studies on the development of guideline-recommended models.

## 2 Materials and methods

This study was reported following the Preferred Reporting Items for Systematic Review and Meta-analysis (PRISMA) (Supplementary Table 1) (26).

### 2.1 Identification of eligible guidelines

We focused on searching the CPGs that recommend any CVD prediction models for clinical practice. Relevant guidelines published in English were searched in the following electronic databases on 28 December 2023: Medline (PubMed), EMBASE, and Web of Science. Considering the impact of the timeliness of the CPGs on their clinical utility, the publication date was restricted to after 2018. The search strategy was developed by a combination of Medical Subject Heading terms as well as text words related to the concepts of “cardiovascular diseases” and “practice guideline” (Supplementary Table 2). Furthermore, the following websites of governments or organizations were used for the supplementary search: World Health Organization (WHO, https://www.who.int/zh/publications), Guidelines International Network (GIN, https://g-i-n.net/), National Institute for Health and Clinical Excellence (NICE, https://www.nice.org.uk/), Scottish Intercollegiate Guidelines Network (SIGN, https://www.sign.ac.uk/), and Canadian Medical Association: Clinical Practice Guideline (CMACPG, https://www.cma.ca/). In addition, we also examined the references of the documents obtained through the aforementioned search strategies to further discover potentially eligible CPGs.

Eligible publications needed to provide a specific recommendation (whether in support or non-support) for at least one multivariable clinical prediction model that was defined as any combination of two or more predictors (variables, features) for estimating the probability or risk of an individual having (diagnosis) or developing (prognosis) a particular outcome in the cardiovascular domain. Guidelines were excluded if they were ongoing, publicly unpublished, or unavailable for full text. In addition, the older versions of guidelines developed by the same academic organization were also excluded in the screening stage. The procedure for identifying eligible guidelines was conducted according to the PRISMA flow diagram (27).

### 2.2 Screening process

The literature search was conducted by one reviewer (C-yJ) after consulting an experienced librarian who also assisted in developing the search strategy. All the search results were imported into Endnote V20.6 to manage the records and screen the eligible CPGs. Two reviewers (C-yJ and LZ) independently screened the titles and abstracts of all the available records. A list of potentially eligible publications was generated for the retrieval of the full texts. Thereafter, the same reviewers independently continued to assess the eligibility of these publications and determined the final included guidelines. The reasons for the exclusion of records were required to be noted. Throughout the process, any disagreement between the two reviewers was resolved by discussion until a consensus was reached or by consulting with an experienced third reviewer (XL).

While screening the guidelines, the primary studies on the development of the guideline-recommended prediction models were further identified through an examination of the references, and the full texts of the relevant studies were obtained by manual retrieval to gather the model details.

### 2.3 Data collection and synthesis

Based on our study objectives, we developed a data extraction form for the standard data collection procedure. The extraction form contained four sections: (1) general information of the guideline, including name, year developed or updated, country, location, and publication organization(s); (2) prediction model recommendations, including recommended model, overview of recommendations, classification of recommendations, level or quality of evidence, and criteria for recommendation; (3) characteristics of prediction model, including presentation, setting, predicted outcome(s), outcome type and target population or condition (Supplementary Table 3). A critical appraisal was conducted on all the recommendations in the CPGs and a narrative synthesis was performed to identify themes based on the extracted data.

### 2.4 Risk of bias assessment

For the guideline-recommended models, we used the Prediction model Risk Of Bias ASsessment Tool (PROBAST) to assess the ROB of the primary modeling studies (28, 29). PROBAST contains 20 signaling questions developed based on the Delphi method, which can assess the ROB according to four domains (participants, predictors, outcome, and analysis). The overall ROB was judged to be low, high, or unclear based on an assessment of all the signaling questions.

## 3 Results

**Figure 1:**
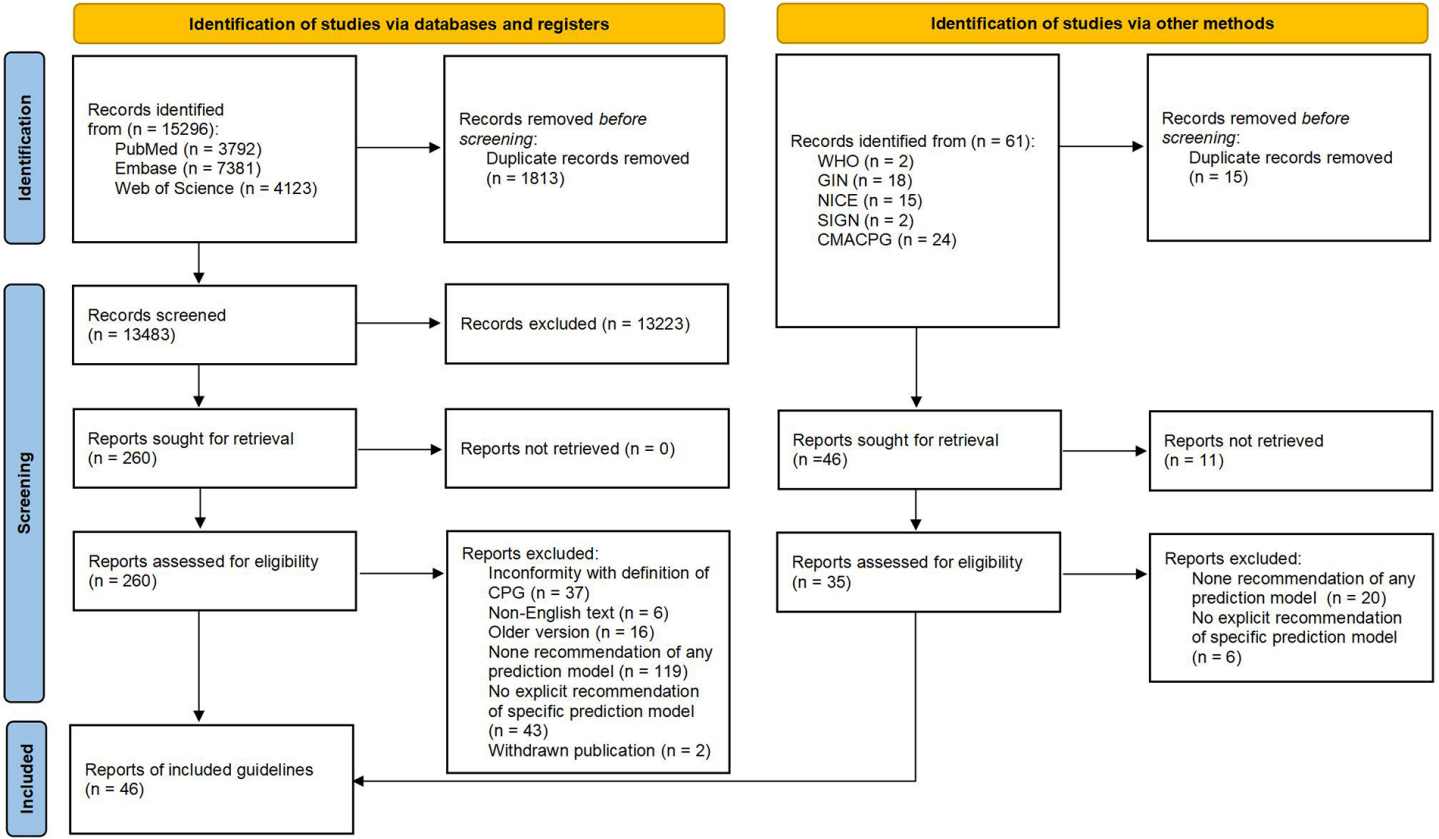
PRISMA flow diagram of the selection of guidelines.

A total of 15,296 potentially relevant records were identified from databases, 13,483 of which were screened based on titles and abstracts after removing 1,813 duplicates. We further identified 260 publications for final eligibility by assessing their full text. Of these, 37 guidelines (3, 12–14, 30–62) fulfill our predefined inclusion criteria while the other 223 publications were excluded for specific reasons (Supplementary Table 4). In addition, we included nine guidelines (16, 63–70) from 61 publications that were identified through the supplementary search. Figure 1 shows the PRISMA flow diagram for the entire CPG selection process.

### 3.1 Characteristics of the included CPGs

In the past 6 years, 46 cardiovascular CPGs with explicit recommendations for clinical prediction models were developed or updated globally. Except for one guideline that was developed by an international academic working group, the other 45 guidelines were published in Europe (19/45, 42%), North America (11/45, 24%), Asia (10/45, 22%), South America (3/45, 7%), and Oceania (2/45, 4%), encompassing 12 countries in total (Supplementary Table 5).

All of these CPGs were evidence-based guidelines developed based on six categories of criteria for forming recommendations. The Grading of Recommendations Assessment, Development and Evaluation (GRADE) approach and a modified GRADE approach (17/46, 37%) were most widely adopted for assessing the quality of evidence and grading recommendations, with the European Society of Cardiology (ESC) approach (15/46, 33%) and the American College of Cardiology Foundation (ACCF)/AHA approach (11/46, 24%) following closely. The American Physical Therapy Association (APTH) approach, European Society for Medical Oncology (ESMO) approach, and Traffic light signaling system-based classification were used only once (1/46, 2%), respectively. The details of all the criteria are summarized in Supplementary Table 6.

### 3.2 Overview of recommendations on prediction models

**Figure 2:**
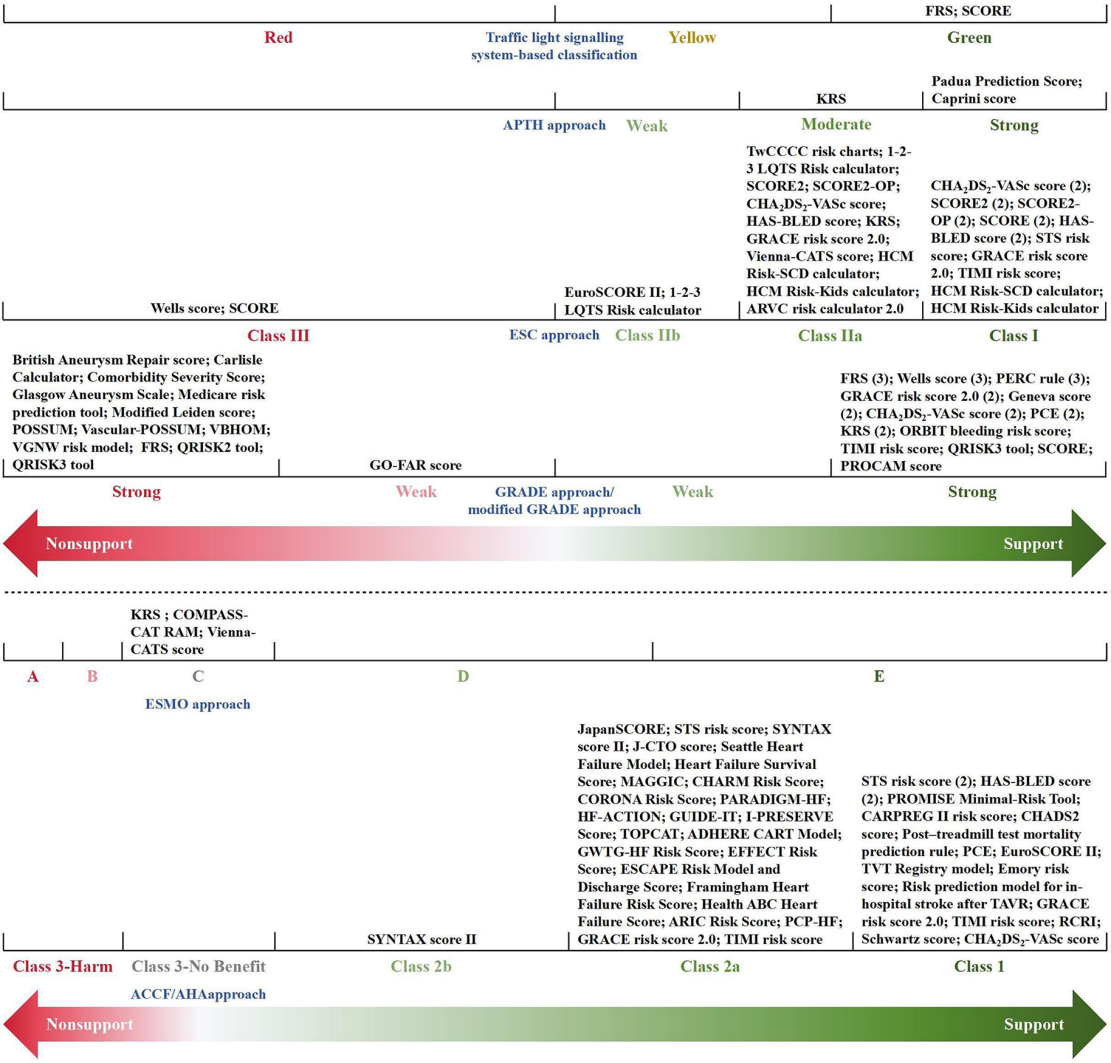
Classification of recommendations for prediction models. The numbers in parentheses represent the sum total of the guidelines with the same classification of recommendation for a specific model. AHA, aortic dissection risk score; ADHERE, acute decompensated heart failure national registry; ARIC, atherosclerosis risk in communities; ARVC, arrhythmogenic right ventricular cardiomyopathy; CARPREG, cardiac disease in pregnancy study; CART, classification and regression tree; CHADS2, congestive heart failure, hypertension, age >75 years, diabetes, stroke/transient ischemic attack; CHA_2_DS_2_-VASc, congestive heart failure, hypertension, age ≥75 years (2 points), diabetes mellitus, stroke (2 points)-vascular disease, age 65–74 years, sex category (female); CHARM, candesartan in heart failure-assessment of reduction in mortality and morbidity; COMPASS-CAT, prospective comparison of methods for thromboembolic risk assessment with clinical perceptions and awareness in real-life patients-cancer associated thrombosis; CORONA, controlled rosuvastatin multinational trial in heart failure; EFFECT, enhanced feedback for effective cardiac treatment; ESCAPE, evaluation study of congestive heart failure and pulmonary artery catheterization effectiveness; FRS, Framingham risk score; GO-FAR, good outcome following attempted resuscitation; GRACE, global registry of acute coronary events; GUIDE-IT, guiding evidence-based therapy using biomarker intensified treatment; GWTG-HF, get with the guidelines—heart failure; HAS-BLED, hypertension, abnormal renal/liver function, stroke, bleeding history or predisposition, labile international normalized ratio, elderly (>65 years), drugs/alcohol concomitantly; HCM, hypertrophic cardiomyopathy; HF, heart failure; HF-ACTION, heart failure: a controlled trial investigating outcomes of exercise training; HFSA, Heart Failure Society of America; I-PRESERVE, irbesartan in heart failure with preserved ejection fraction study; J-CTO, multicenter chronic total occlusion registry in Japan; KRS, Khorana risk score; LQTS, long QT syndrome; MAGGIC, meta-analysis global group in chronic heart failure; PARADIGM-HF, prospective comparison of ARNI with ACEI to determine impact on global mortality and morbidity in heart failure trial; PCE, pooled cohort equations; PCP-HF, pooled cohort equations to prevent HF; PERC, pulmonary embolism rule-out criteria; POSSUM, physiological and operative severity score for enumeration of mortality; PROCAM, prospective cardiovascular Münster; PROMISE, prospective multicenter imaging study for evaluation of chest pain; RAM, risk assessment model; RCRI, revised cardiac risk index; SCD, sudden cardiac death; SCORE, systematic coronary risk estimation; SCORE2, systematic coronary risk estimation 2; SCORE2-OP, systematic coronary risk estimation 2-older persons; STS, Society of Thoracic Surgeons; SYNTAX, synergy between percutaneous coronary intervention with TAXUS and cardiac surgery; TAVR, transcatheter aortic valve replacement; TIMI, thrombolysis in myocardial infarction; TOPCAT, treatment of preserved cardiac function heart failure with an aldosterone antagonist trial; TVT, transcatheter valve therapy; TwCCCC, Taiwan Chin-Shan community cardiovascular cohort; VBHOM, vascular biochemical and haematological outcome model; VGNW, vascular governance North West; Vienna-CATS, Vienna cancer and thrombosis study.

A total of 80 eligible recommendations and 69 relevant prediction models were identified and extracted from the included CPGs. An overview of the recommendations is given in Supplementary Table 7. Of the 80 recommendations, 74 (93%) supported the application of an appropriate prediction model in clinical practice, while the other 6 (7%) explicitly put forward a disapproving opinion of the specific model. Figure 2 synthesizes and visualizes the recommendation classifications of the 69 models based on different criteria. Thus, the involved models could be divided into three categories: (1) 53 fully recommended models (only mentioned in the supportive recommendations); (2) 4 conditionally recommended models (mentioned in both the supportive and non-supportive recommendations); and (3) 12 non-recommended models (only mentioned in the non-supportive recommendations). More importantly, for recommendations regarding the use of prognostic models, 10 CPGs recommended 16 prognostic models for further clinical decision-making or treatment selection besides risk assessment.

**Figure 3:**
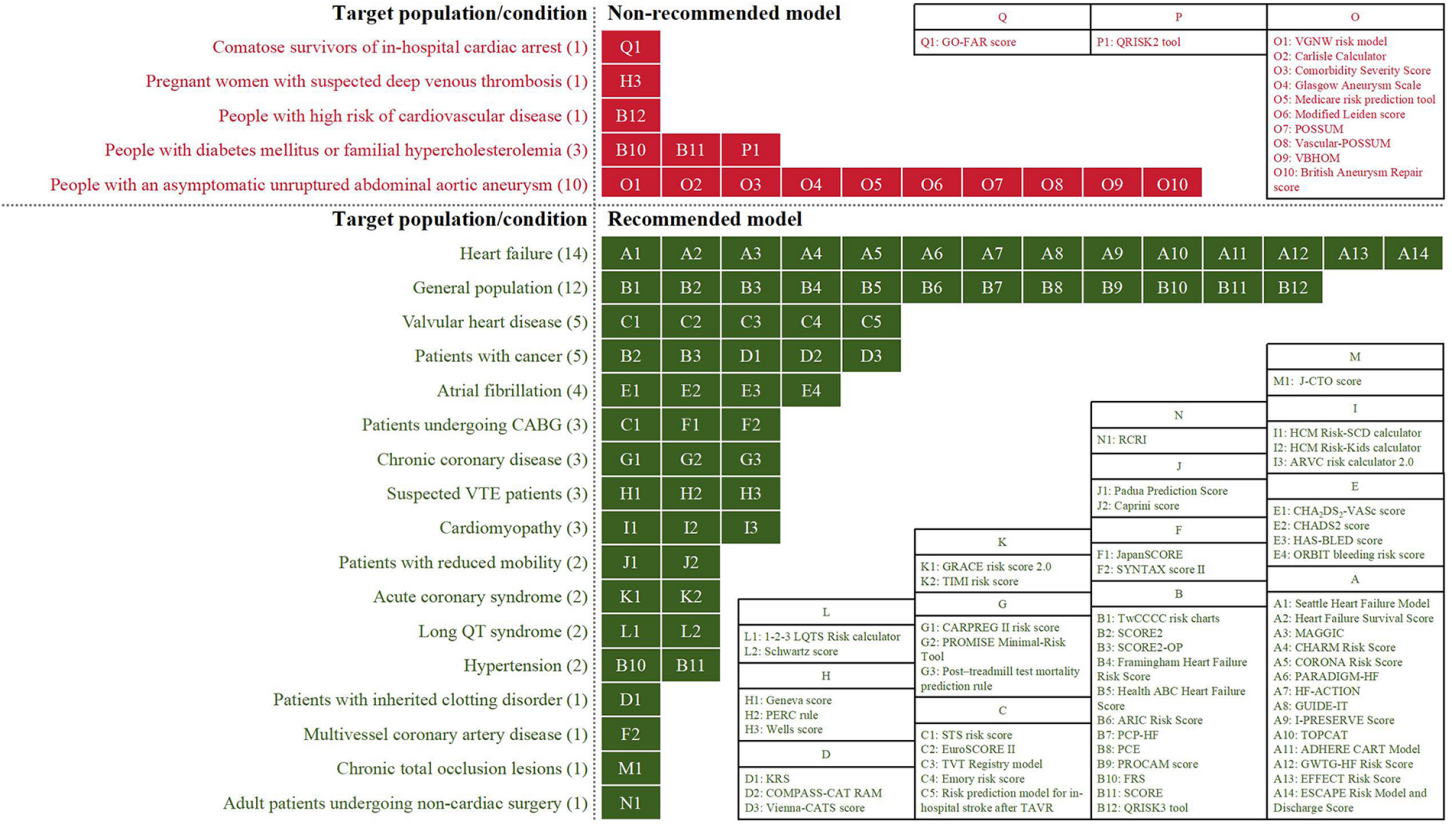
The recommended and non-recommended target populations/conditions in clinical practice of the prediction models.

Due to different evidence-grading criteria, there was wide variation in the level/quality of evidence for supporting the application of a model. However, almost all guideline-recommended (both fully recommended and conditionally recommended) models had a moderate to strong strength of recommendation, of which the conditionally recommended models were discouraged for populations with specific known risk factors (e.g., diabetes mellitus and familial hypercholesterolemia). Meanwhile, based on the low to moderate levels of evidence, the guidelines discouraged the use of all of the non-recommended models in unsuited target populations (e.g., pregnant women with suspected deep vein thrombosis) or conditions (e.g., in-hospital cardiac arrest). Figure 3 summarizes the recommended and non-recommended target populations/conditions in clinical practice of the three categories of models.

### 3.3 Attributes of the guideline-recommended models in cardiovascular practice

Table 1 shows the attributes of the guideline-recommended models in cardiovascular practice. Almost all the recommended models were prognostic models (53/57, 93%) and were focused on primary (16/57, 28%) and tertiary prevention (37/57, 65%), while the remaining models (4/57, 7%) were diagnostic models and were focused on secondary prevention.

**Table 1:** Attributes of the guideline-recommended models (*N* = 57 models).

Attribute	Total, *n* (%)
Outcome type	
Prognostic	53 (93)
Diagnostic	4 (7)
Clinical setting	
Primary prevention	16 (28)
Secondary prevention	4 (7)
Tertiary prevention	37 (65)
Target condition/population	
Heart failure	14 (25)
General population with cardiovascular risk factor(s) or without	12 (21)
Valvular heart disease	5 (9)
Patients with cancer	5 (9)
Atrial fibrillation	4 (7%)
Chronic coronary disease	3 (5)
Cardiomyopathy	3 (5)
Suspected venous thromboembolism patients (including deep venous thrombosis and pulmonary embolism)	3 (5)
Patients undergoing CABG	3 (5)
Acute coronary syndrome	2 (4)
Long QT syndrome	2 (4)
Hypertension	2 (4)
Patients with reduced mobility	2 (4)
Multivessel coronary artery disease	1 (2)
Chronic total occlusion lesions	1 (2)
Patients with inherited clotting disorder	1 (2)
Adult patients undergoing non-cardiac surgery	1 (2%
Predicted outcome(s)	
Risk of mortality	23 (40)
Risk of incident fatal or non-fatal cardiovascular disease	8 (14)
Risk of incident venous thromboembolism	5 (9)
Risk of incident heart failure	4 (7)
Pretest probability of incident acute pulmonary embolism	3 (5)
Risk of incident major bleeding	2 (4)
Risk of incident major adverse cardiovascular events	2 (4)
Risk of incident stroke	2 (4)
Risk of incident cardiac complications	1 (2)
Risk of incident life-threatening arrhythmias	1 (2)
Risk of incident major cardiac complications after non-cardiac surgery	1 (2)
Pretest probability of incident long QT syndrome	1 (2)
Pretest probability of successful PCI	1 (2)
Pretest probability of the need for PMI after TAVR	1 (2)
Risk of incident stroke or systemic thromboembolism	1 (2)
Risk of mortality or incident myocardial infarction	1 (2)
Presentation format	
Points score system	29 (51)
Website calculator	14 (25)
Nomogram	2 (4)
Graphical score chart	1 (2)
Points score system combined with website	4 (7)
Graphical score chart combined with website	3 (5)
Points score system combined with nomogram	1 (2)
Unclear	3 (5)

CABG, coronary artery bypass grafting; PCI, percutaneous coronary intervention; PMI, pacemaker implantation; TAVR, transcatheter aortic valve replacement.

A total of 10 conditions and seven types of target population were involved in the 57 models, where HF (14/57, 25%) and a general population with or without cardiovascular risk factor(s) (12/57, 21%) had received the most attention from the guidelines. Other target conditions included valvular heart disease (5/57, 9%), atrial fibrillation (4/57, 7%), cardiomyopathy (3/57, 5%), chronic coronary disease (3/57, 5%), acute coronary syndrome (2/57, 4%), long QT syndrome (2/57, 4%), hypertension (2/57, 4%), multivessel coronary artery disease (1/57, 2%), and chronic total occlusion lesions (1/57, 2%). Suspected venous thromboembolism patients (3/57, 5%); patients who had undergone coronary artery bypass grafting (CABG) (3/57, 5%) or non-cardiac surgery (1/57, 2%); or patients with cancer (5/57, 9%), reduced mobility (2/57, 4%), or an inherited clotting disorder (1/57, 2%) were also target populations.

The predicted outcome of the recommended models was mostly concerned with the risk of mortality (23/57, 40%), followed by the risk of incident fatal or non-fatal CVD (8/57, 14%) and the risk of incident venous thromboembolism (5/57, 9%). Others mainly focused on predicting the risk of incident cardiovascular complications and evaluating the feasibility/necessity of the interventions under consideration. A point scoring system (29/57, 51%) was the most common presentation format of the prediction models for use in clinical settings, followed by a website calculator (14/57, 25%). A nomogram (2/57, 4%) or graphical score chart (1/57, 2%) was relatively rare. In addition, the presentation of eight (14%) models combined two of the above four formats, while the remaining three (5%) models were unclear.

### 3.4 Risk of bias assessment of the primary modeling studies

**Figure 4:**
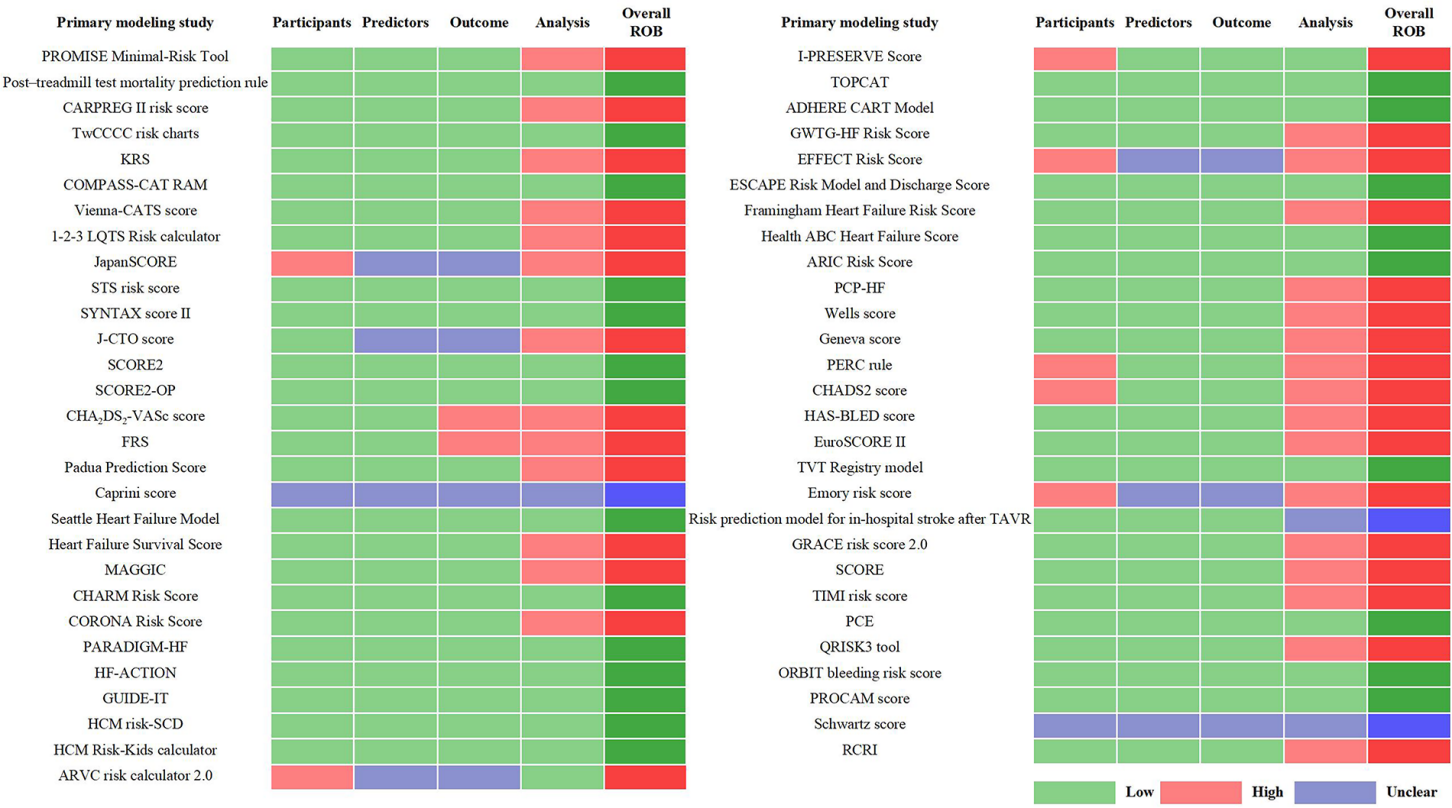
ROB of each primary modeling study.

**Figure 5:**
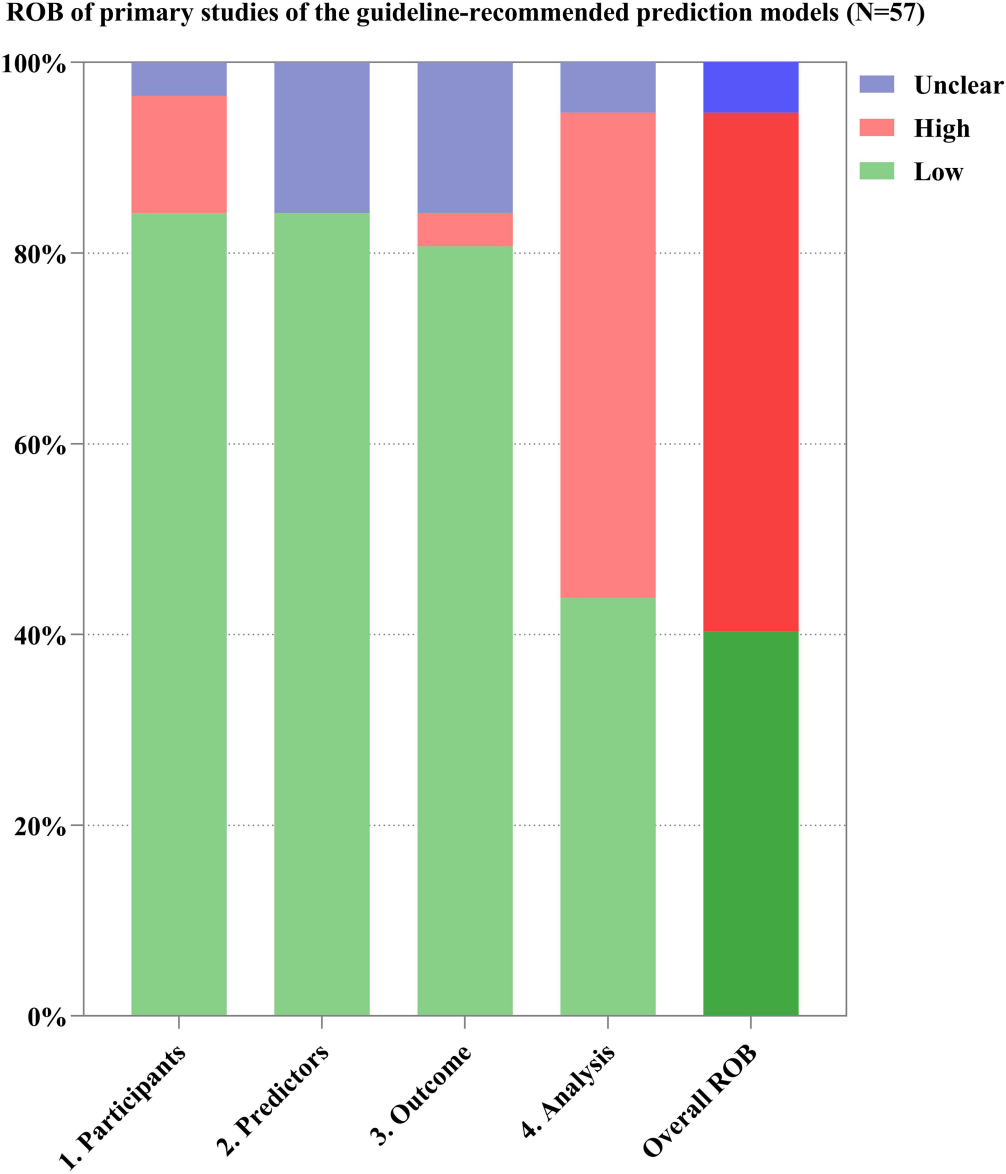
ROB of the primary studies on guideline-recommended prediction models.

Through the references provided in the guidelines and from manual retrieval, we further identified 57 primary studies on the development of the guideline-recommended models (Supplementary Table 8). Of the 57 primary studies on the guideline-recommended prediction models, 23 (40%) studies were rated as having a low ROB, 3 (4%) as having an unclear ROB, and the remaining 31 (56%) studies had a high ROB after PROBAST analysis (Figures 4, 5). The details of the ROB assessment of 20 signaling questions are shown in Supplementary Table 9.

The causes of high ROB were mainly in the analysis domain as 23 (40%) studies inappropriately evaluated the calibration of the relevant models (e.g., ignored the calibration or evaluated the calibration using the Hosmer–Lemeshow test only) and 17 (30%) studies did not account for the fitting of their models (e.g., a lack of internal validation or the dataset was randomly split into only derivation and validation sets). Other reasons for a high ROB included incorrect handling in continuous/categorical predictors and improper variable selection (i.e., based only on univariable analysis). A non-prospective data source was the major cause of high ROB in the participant domain, resulting in six (11%) studies being rated as having a high ROB. This also led to an unclear ROB in the blinding of predictor or outcome assessment with regard to the reporting of relevant missing content in such studies. Beyond that, there was no information about handling missing data and consistency in reporting the predictor weighting in 11 (19%) and 8 (14%) studies, respectively, leading to an unclear ROB for the relevant signaling questions.

## 4 Discussion

In this global survey of 46 qualified cardiovascular CPGs involving 80 recommendations for 69 relevant prediction models from 2018 to 2023, we found that 74 recommendations supported 57 models in cardiovascular practice with moderate to strong strength. Most of the guideline-recommended models were focused on predicting prognosis outcomes in primary and tertiary prevention, mainly concentrating on long-term risk stratification and prognosis management. Moreover, a further assessment of the methodological quality of the primary studies on the development of the guideline-recommended models revealed that only 40% of the modeling studies had a low risk of bias.

The findings of this study indicate that prediction models have been generally recommended for cardiovascular practice in global CPGs, although their primary application was concentrated in developed countries in Europe and the Americas, geographically. Given the abundance of developed prediction models and the rapidly increasing number of similar modeling studies, how to evaluate and recommend models with genuine clinical utility still remains a major dilemma for guideline creators and methodologists. Indeed, a phenomenon—the overall level of evidence for the recommendations of moderate to strong strength was moderate or even lower—emerged in this study. It is common for a recommendation of strong strength to only be supported by consensus/expert opinion-level evidence (41, 46, 50, 52). This may be attributed to the stability (via internal validation) and generalizability (via external validation) of the validated models despite the paucity of high-level evidence (71).

With regards to evidence-based CPGs, evidence is the cornerstone of formulating recommendations, and high-level evidence is universally defined as results from high-quality randomized controlled trials (RCTs) or meta-analyses (24). However, when conducting RCTs or meta-analyses for prediction models that are treated as specific interventions, additional challenges need to be considered more carefully. First, the applicable study design for investigating the impact of models on cardiovascular practice is a cluster randomized trial (CRT) (72, 73). Compared to the conventional RCT design, a CRT requires a considerably larger sample size for a statistically significant effect size and this sometimes faces several particular medical ethical challenges, such as the plausibility of informed consent waivers and the possible neglect of vulnerable individuals (74, 75). Second, the availability and heterogeneity of the original data, mainly resulting from reporting and selection biases, may hinder the synthesis of evidence in meta-analyses (29). Third, there are no established indicators or criteria that can be used to make a fully objective distinction between the superiority or inferiority of a model based on the results of meta-analyses, despite the use of the most common methods of discrimination such as the area under the receiver operating characteristic curve (AUC). But in view of poor methodological quality and ubiquitous high ROB, almost all the systematic reviews with or without a meta-analysis have adopted a deliberative attitude toward the application of prediction models in cardiovascular practice, regardless of their discrimination (76, 77). Addressing the aforementioned challenges will be critical for obtaining more high-level evidence in the future. More importantly, in terms of the prediction models themselves, how and when to update the existing models have been problems in an increasingly complex real-world medical setting, especially for certain specific cardiovascular diseases where treatment modalities and drugs are rapidly evolving. Thus, for static models (also called time-independent models), it is crucial to address calibration drift to maintain effective and efficient performance when the treatment is part of the predictors (78). Correspondingly, due to the lag inherent to the CPGs themselves, it will be wise and prudent for recommendations to take into account information regarding the updating (including frequency, aim, and methods of updating) of the recommended prediction model.

We also observed that the same models were recommended for heterogeneous target populations or conditions in different cardiovascular CPGs. For example, in addition to the general population (14), cancer patients treated with endocrine therapies were another target population recommended for the application of SCORE2 in the 2022 ESC Guidelines on cardio-oncology (41). Similar examples included the FRS (for the general population or patients with hypertension) (13, 54), Khorana risk score (for patients with cancer or inherited clotting disorder) (34, 42), and Society of Thoracic Surgeons risk score (for patients undergoing CABG or with valvular heart disease) (40, 46). Such excellent generalizability via external validation indicates the great application potential of a single well-established prediction model, which may contribute to research resource savings by reducing superfluous modeling studies (79).

To the best of our knowledge, this study was the first to systematically synthesize the recommendations for prediction models in cardiovascular CPGs published in the last 5 years while also conducting a methodological quality assessment of the primary studies on the development of the guideline-recommended models. The results may be of referential value to both clinicians and guideline methodologists. For future studies, substantial efforts are still required to improve the methodological quality of clinical prediction models, as well as their extensive external validation.

The limitations of this study merit further consideration. First, in consideration of the particularity of CPGs in terms of timeliness, we only included cardiovascular CPGs from 2018 to 2023, possibly omitting qualified guidelines that were published before 2018 and had an update period longer than 6 years. As a result, the few early-developed prediction models still in use may not have been assessed in this study. Second, in the case of the primary studies on the development of the guideline-recommended models identified through the references provided in the guidelines and from manual retrieval, there was no guaranteed consistency in model version in the 57 primary studies used for the assessment of methodological quality. This is due to individual models being updated over time, with multiple versions of primary modeling studies. Third, we did not further explore the methodological differences between the recommended and non-recommended models as these were not a major factor that supported or discouraged their use to provide clinical decision-making support. Finally, given the heterogeneous criteria for forming recommendations, we did not analyze the relevance between the strength of recommendation and the overall ROB of the prediction models. Thus, it is difficult to determine what role the ROB plays in the process of recommending models for cardiovascular practice. In any case, a high ROB implies that the performance of these models in new samples will probably be worse than that in the modeling samples, which could lead to improper clinical decisions and thus compromise the benefit for patients in healthcare settings (18, 80).

## 5 Conclusion

Global cardiovascular CPGs presented an unduly positive appraisal of the existing prediction models in terms of ROB, leading to stronger recommendations than were warranted. Future cardiovascular practice may benefit from well-established clinical prediction models with better methodological quality and extensive external validation.

## Data Availability

The original contributions presented in the study are included in the article/Supplementary Material, further inquiries can be directed to the corresponding author.
