# Supplementary material for: Recommendations for prediction models in clinical practice guidelines for cardiovascular diseases are over-optimistic: a global survey utilizing a systematic literature search
Source: Front Cardiovasc Med. 2024 Oct 17;11:1449058. doi: 10.3389/fcvm.2024.1449058 (PMC11524858; doi:10.3389/fcvm.2024.1449058)
Supplement: Supplementary file 1 [file Table1.pdf]

Supplementary Material

Supplementary Table 1 PRISMA 2020 Checklist

Supplementary Table 2 Search Strategy Conducted on December 28, 2023

Supplementary Table 3 Data Extraction Form for Standard Data-collection Procedure

Supplementary Table 4 Guidelines Excluded on Full-text with Reason

Supplementary Table 5 Characteristic of Included CPGs

Supplementary Table 6 Criteria for Forming Recommendations in Included CPGs

Supplementary Table 7 Overview of Recommendations on Prediction Models

Supplementary Table 8 Primary Studies of Guideline-recommended Prediction Models

Supplementary Table 9 Details of ROB Assessment in 20 Signaling Questions of PROBAST

Supplementary Table 1 PRISMA 2020 Checklist

Section and Topic	Item #	Checklist item	Page No
**TITLE**			
Title	1	Identify the report as a systematic review.	
**ABSTRACT**			
Abstract	2	See the PRISMA 2020 for Abstracts checklist.	
**INTRODUCTION**			
Rationale	3	Describe the rationale for the review in the context of existing knowledge.	
Objectives	4	Provide an explicit statement of the objective(s) or question(s) the review addresses.	
**METHODS**			
Eligibility criteria	5	Specify the inclusion and exclusion criteria for the review and how studies were grouped for the syntheses.	
Information sources	6	Specify all databases, registers, websites, organisations, reference lists and other sources searched or consulted to identify studies. Specify the date when each source was last searched or consulted.	
Search strategy	7	Present the full search strategies for all databases, registers and websites, including any filters and limits used.	
Selection process	8	Specify the methods used to decide whether a study met the inclusion criteria of the review, including how many reviewers screened each record and each report retrieved, whether they worked independently, and if applicable, details of automation tools used in the process.	
Data collection process	9	Specify the methods used to collect data from reports, including how many reviewers collected data from each report, whether they worked independently, any processes for obtaining or confirming data from study investigators, and if applicable, details of automation tools used in the process.	
Data items	10a	List and define all outcomes for which data were sought. Specify whether all results that were compatible with each outcome domain in each study were sought (e.g. for all measures, time points, analyses), and if not, the methods used to decide which results to collect.	
	10b	List and define all other variables for which data were sought (e.g. participant and intervention characteristics, funding sources). Describe any assumptions made about any missing or unclear information.	
Study risk of bias assessment	11	Specify the methods used to assess risk of bias in the included studies, including details of the tool(s) used, how many reviewers assessed each study and whether they worked independently, and if applicable, details of automation tools used in the process.	
Effect measures	12	Specify for each outcome the effect measure(s) (e.g. risk ratio, mean difference) used in the synthesis or presentation of results.	
Synthesis methods	13a	Describe the processes used to decide which studies were eligible for each synthesis (e.g. tabulating the study intervention characteristics and comparing against the planned groups for each synthesis (item #5)).	
	13b	Describe any methods required to prepare the data for presentation or synthesis, such as handling of	

		missing summary statistics, or data conversions.	
	13c	Describe any methods used to tabulate or visually display results of individual studies and syntheses.	
	13d	Describe any methods used to synthesize results and provide a rationale for the choice(s). If meta-analysis was performed, describe the model(s), method(s) to identify the presence and extent of statistical heterogeneity, and software package(s) used.	
	13e	Describe any methods used to explore possible causes of heterogeneity among study results (e.g. subgroup analysis, meta-regression).	
	13f	Describe any sensitivity analyses conducted to assess robustness of the synthesized results.	
Reporting bias assessment	14	Describe any methods used to assess risk of bias due to missing results in a synthesis (arising from reporting biases).	
Certainty assessment	15	Describe any methods used to assess certainty (or confidence) in the body of evidence for an outcome.	
**RESULTS**			
Study selection	16a	Describe the results of the search and selection process, from the number of records identified in the search to the number of studies included in the review, ideally using a flow diagram.	
	16b	Cite studies that might appear to meet the inclusion criteria, but which were excluded, and explain why they were excluded.	
Study characteristics	17	Cite each included study and present its characteristics.	
Risk of bias in studies	18	Present assessments of risk of bias for each included study.	
Results of individual studies	19	For all outcomes, present, for each study: (a) summary statistics for each group (where appropriate) and (b) an effect estimate and its precision (e.g. confidence/credible interval), ideally using structured tables or plots.	
Results of syntheses	20a	For each synthesis, briefly summarise the characteristics and risk of bias among contributing studies.	
	20b	Present results of all statistical syntheses conducted. If meta-analysis was done, present for each the summary estimate and its precision (e.g. confidence/credible interval) and measures of statistical heterogeneity. If comparing groups, describe the direction of the effect.	
	20c	Present results of all investigations of possible causes of heterogeneity among study results.	
	20d	Present results of all sensitivity analyses conducted to assess the robustness of the synthesized results.	
Reporting biases	21	Present assessments of risk of bias due to missing results (arising from reporting biases) for each synthesis assessed.	
Certainty of evidence	22	Present assessments of certainty (or confidence) in the body of evidence for each outcome assessed.	
**DISCUSSION**			
Discussion	23a	Provide a general interpretation of the results in the context of other evidence.	
	23b	Discuss any limitations of the evidence included in the review.	

	23c	Discuss any limitations of the review processes used.	
	23d	Discuss implications of the results for practice, policy, and future research.	
**OTHER INFORMATION**			
Registration and protocol	24a	Provide registration information for the review, including register name and registration number, or state that the review was not registered.	
	24b	Indicate where the review protocol can be accessed, or state that a protocol was not prepared.	
	24c	Describe and explain any amendments to information provided at registration or in the protocol.	
Support	25	Describe sources of financial or non-financial support for the review, and the role of the funders or sponsors in the review.	
Competing interests	26	Declare any competing interests of review authors.	
Availability of data, code and other materials	27	Report which of the following are publicly available and where they can be found: template data collection forms; data extracted from included studies; data used for all analyses; analytic code; any other materials used in the review.	

Supplementary Table 2 Search Strategy Conducted on December 28, 2023

Source	Search strategy	Records
PubMed	(("cardiovascular diseases"[MeSH Terms] OR "cardiovascular abnormalities"[MeSH Terms] OR "cardiovascular infections"[MeSH Terms] OR "heart diseases"[MeSH Terms] OR "pregnancy complications, cardiovascular"[MeSH Terms] OR "vascular diseases"[MeSH Terms] OR ("cardiovascular disease*"[Title/Abstract] OR "cardiovascular disorder*"[Title/Abstract] OR "cardiovascular event*"[Title/Abstract] OR "coronary disease*"[Title/Abstract] OR "coronary event*"[Title/Abstract] OR "coronary disorder*"[Title/Abstract] OR "peripheral vascular disease*"[Title/Abstract] OR "peripheral vascular event*"[Title/Abstract] OR "peripheral vascular disorder*"[Title/Abstract] OR "heart disease*"[Title/Abstract] OR "heart event*"[Title/Abstract] OR "heart disorder*"[Title/Abstract] OR "peripheral arterial disease*"[Title/Abstract] OR "peripheral arterial event*"[Title/Abstract] OR "peripheral arterial disorder*"[Title/Abstract] OR "heart death*"[Title/Abstract] OR "heart arrest*"[Title/Abstract] OR "heart attack*"[Title/Abstract] OR "cardiopulmonary death*"[Title/Abstract] OR "cardiopulmonary arrest*"[Title/Abstract] OR ("cardiopulmonary"[All Fields] AND "attack*"[Title/Abstract]) OR "cardiac death*"[Title/Abstract] OR "cardiac arrest*"[Title/Abstract] OR "cardiac attack*"[Title/Abstract] OR "hypertension"[Title/Abstract] OR "hypertensive*"[Title/Abstract] OR "atheroscleros*"[Title/Abstract] OR "arterioscleros*"[Title/Abstract] OR "myocardial infarct*"[Title/Abstract] OR "acute coronary syndrome*"[Title/Abstract] OR "atrial fibrillation"[Title/Abstract] OR "heart failure"[Title/Abstract] OR "CVD"[Title/Abstract] OR "CHD"[Title/Abstract] OR "CAD"[Title/Abstract] OR "PAD"[Title/Abstract] OR "CVA"[Title/Abstract] OR "MI"[Title/Abstract] OR "ACS"[Title/Abstract] OR "AF"[Title/Abstract] OR "HF"[Title/Abstract])) AND ("practice guideline"[Publication Type] OR "practice guideline"[Title/Abstract] OR "clinical practice guideline*"[Title/Abstract] OR "clinical guideline*"[Title/Abstract]) AND 2018/01/01:2023/12/31[Date - Publication]) AND (english[Filter])	3792
Embase	('cardiovascular disease'/exp OR 'coronary artery disease'/exp OR 'vascular disease'/exp OR 'coronary atherosclerosis'/exp OR 'peripheral vascular disease'/exp OR 'peripheral arterial disease'/exp OR 'arteriosclerosis'/exp OR 'coronary occlusion'/exp OR 'hypertension'/exp OR 'heart disease'/exp OR 'heart arrhythmia'/exp OR 'heart fibrillation'/exp OR 'atrial fibrillation'/exp OR 'heart failure'/exp OR 'acute coronary syndrome'/exp OR 'angina pectoris'/exp OR 'heart infarction'/exp OR 'heart arrest'/exp OR 'heart death'/exp OR 'atherosclerosis'/exp OR 'cardiovascular disease*':ab,ti OR 'cardiovascular disorder*':ab,ti OR 'cardiovascular event*':ab,ti OR 'coronary disease*':ab,ti OR 'coronary event*':ab,ti OR 'coronary disorder*':ab,ti OR 'peripheral vascular disease*':ab,ti OR 'peripheral vascular event*':ab,ti OR 'peripheral vascular disorder*':ab,ti OR 'heart disease*':ab,ti OR 'heart event*':ab,ti OR 'heart disorder*':ab,ti OR 'peripheral arterial disease*':ab,ti OR 'peripheral arterial event*':ab,ti OR 'peripheral arterial disorder*':ab,ti OR 'heart death*':ab,ti OR 'heart arrest*':ab,ti OR 'heart attack*':ab,ti OR 'cardiopulmonary death*':ab,ti OR 'cardiopulmonary arrest*':ab,ti OR 'cardiopulmonary attack*':ab,ti OR 'cardiac death*':ab,ti OR 'cardiac	7381

	arrest*':ab,ti OR 'cardiac attack*':ab,ti OR hypertension:ab,ti OR hypertensive*:ab,ti OR atheroscleros*:ab,ti OR arterioscleros*:ab,ti OR 'myocardial infarct*':ab,ti OR 'acute coronary syndrome*':ab,ti OR 'atrial fibrillation':ab,ti OR 'heart failure':ab,ti OR cvd:ab,ti OR chd:ab,ti OR cad:ab,ti OR pad:ab,ti OR cva:ab,ti OR mi:ab,ti OR acs:ab,ti OR af:ab,ti OR hf:ab,ti) AND 'practice guideline'/mj/exp AND ('practice guideline*':ti OR 'clinical practice guideline*':ti OR 'clinical guideline*':ti OR 'guideline*':ti) AND (2018:py OR 2019:py OR 2020:py OR 2021:py OR 2022:py OR 2023:py) AND [english]/lim	
Web of Science	(((TS=(cardiovascular disease*)) OR TS=(cardiovascular disorder*)) OR TS=(cardiovascular event*)) OR TS=(coronary disease*)) OR TS=(coronary event*)) OR TS=(coronary disorder*)) OR TS=(vascular disease*)) OR TS=(vascular event*)) OR TS=(vascular disorder*)) OR TS=(heart disease*)) OR TS=(heart event*)) OR TS=(heart disorder*)) OR TS=(arterial disease*)) OR TS=(arterial event*)) OR TS=(arterial disorder*)) OR TS=(cardiovascular abnormalit*)) OR TS=(cardiovascular infection*)) OR TS=(heart death*)) OR TS=(heart arrest*)) OR TS=(heart attack*)) OR TS=(cardiopulmonary death*)) OR TS=(cardiopulmonary arrest*)) OR TS=(cardiopulmonary attack*)) OR TS=(cardiac death*)) OR TS=(cardiac arrest*)) OR TS=(cardiac attack*)) OR TS=(hypertension)) OR TS=(hypertensive*)) OR TS=(atheroscleros*)) OR TS=(arterioscleros*)) OR TS=(myocardial infarct*)) OR TS=(acute coronary syndrome*)) OR TS=(atrial fibrillation)) OR TS=(heart failure)) OR TS=(CVD)) OR TS=(CHD)) OR TS=(CAD)) OR TS=(PAD)) OR TS=(CVA)) OR TS=(MI)) OR TS=(ACS)) OR TS=(AF)) OR TS=(HF)) AND (((TS=(practice guideline*)) OR TS=(clinical practice guideline*)) OR TS=(clinical guideline*)) AND ((MH=("PRACTICE GUIDELINES AS TOPIC") AND LA=("ENGLISH") AND (PY=(2018 OR 2019 OR 2020 OR 2021 OR 2022 OR 2023))) NOT (SILOID=("PPRN")))	4123

Supplementary Table 3 Data Collection Form for standard data-collection procedure

**Extracted Item**		**Comments**
**General information of the publication**	Name	name of guideline
	Year	published or last updated year of guideline
	Author	authors of guideline
	Country/Location	which country and (or) location was the guideline published?
	Organization(s)	which organization(s) developed the guideline?
**Recommendations of prediction model**	Recommended model	official name of the recommended prediction model, e.g., ARIC Risk Score (Atherosclerosis Risk in Communities Risk Score)
	Overview of recommendations	original text (whether support or nonsupport) based on guideline
	Class of recommendations	also called strength of recommendations, which defined by guideline, e.g., Class I to III
	Quality of evidence	also called level of evidence, which defined by guideline, e.g., Level A to D
	Criteria for forming recommendations	which criteria, e.g., GRADE, were used for forming recommendations?
**Characteristics of prediction model**	Presentation	e.g., points score system, graphical score chart, nomogram, website or application
	Setting	e.g., primary care, secondary care or tertiary care
	Predicted outcome(s)	specific future event or specific diagnostic target disease, e.g., individual CVD risk within the next 10 years or presence of aortic disease
	Outcome type	e.g., diagnostic or prognostic
	Target population or condition	e.g., healthy adult in the general population or patients with chronic heart failure

Supplementary Table 4 Guidelines Excluded on Full-text with Reason

**Source**	**Title of Excluded Guideline**	**Reasons for exclusion**
Database	Korean Society of Heart Failure Guidelines for the Management of Heart Failure	None recommendation of any prediction model
Database	International Academy of Perinatal Medicine (IAPM) guidelines for screening, prediction, prevention and management of pre-eclampsia to reduce maternal mortality in developing countries	None recommendation of any prediction model
Database	Saudi Critical Care Society clinical practice guidelines on the prevention of venous thromboembolism in adults with trauma: Endorsement by the Scandinavian Society of Anaesthesiology and Intensive Care Medicine	None recommendation of any prediction model
Database	Joint 2022 European Society of Thoracic Surgeons and the American Association for Thoracic Surgery guidelines for the prevention of cancer-associated venous thromboembolism in thoracic surgery	No explicit recommendation of specific prediction model
Database	The 2023 International Society for Heart and Lung Transplantation Guidelines for Mechanical Circulatory Support: A 10- Year Update	No explicit recommendation of specific prediction model
Database	Practical Guidelines for Patients with Hypertension and Periodontitis	None recommendation of any prediction model
Database	Continuing Medical Education Pediatric Hypertension: An Update on the American Academy of Pediatrics Clinical Practice Guidelines	Withdrawn publication
Database	European Society for Vascular Surgery (ESVS) 2023 Clinical Practice Guidelines on the Management of Atherosclerotic Carotid and Vertebral Artery Disease	No explicit recommendation of specific prediction model
Database	JCS 2023 Guideline on the Diagnosis and Treatment of Myocarditis	None recommendation of any prediction model
Database	American Society of Hematology 2023 Guidelines for Management of Venous Thromboembolism: Thrombophilia Testing	None recommendation of any prediction model
Database	Guidelines for Diagnosis and Management of Infective Endocarditis in Adults: A WikiGuidelines Group Consensus Statement	Inconformity with definition of CPG
Database	2023 ESH Guidelines for the management of arterial hypertension The Task Force for the management of arterial hypertension of the European Society of Hypertension Endorsed by the European Renal Association (ERA) and the International Society of Hypertension (ISH)	None recommendation of any prediction model
Database	The 2022 focused update of the 2018 Korean Hypertension Society Guidelines for the management of hypertension	None recommendation of any prediction model
Database	2022 ACC/AHA Guideline for the Diagnosis and Management of Aortic Disease	No explicit recommendation of specific prediction model
Database	2022 ESC/ERS Guidelines for the diagnosis and treatment of pulmonary hypertension	No explicit recommendation of specific prediction model
Database	JCS/CVIT/JCC 2023 Guideline Focused Update on Diagnosis and Treatment of Vasospastic Angina (Coronary Spastic Angina) and Coronary Microvascular Dysfunction	None recommendation of any prediction model
Database	Cardiac arrest in the perioperative period: a consensus guideline for	None recommendation of any

	identification, treatment, and prevention from the European Society of Anaesthesiology and Intensive Care and the European Society for Trauma and Emergency Surgery	prediction model
Database	Canadian Cardiovascular Society-Canadian Heart Failure Society Focused Clinical Practice Update of Patients With Differing Heart Failure Phenotypes	None recommendation of any prediction model
Database	2023 HRS/APHRS/LAHRS guideline on cardiac physiologic pacing for the avoidance and mitigation of heart failure	None recommendation of any prediction model
Database	Korean Society of Heart Failure Guidelines for the Management of Heart Failure: Definition and Diagnosis	None recommendation of any prediction model
Database	ACR Appropriateness Criteria® Pulsatile Abdominal Mass, Suspected Abdominal Aortic Aneurysm: 2023 Update	Inconformity with definition of CPG
Database	The International Society for Heart and Lung Transplantation (ISHLT) guidelines for the care of heart transplant recipients	No explicit recommendation of specific prediction model
Database	Editor's Choice - European Society for Vascular Surgery (ESVS) 2023 Clinical Practice Guidelines on Antithrombotic Therapy for Vascular Diseases	No explicit recommendation of specific prediction model
Database	2023 World Heart Federation guidelines for the echocardiographic diagnosis of rheumatic heart disease	None recommendation of any prediction model
Database	ACR Appropriateness Criteria® Lower Extremity Chronic Venous Disease	Inconformity with definition of CPG
Database	Korean Society of Heart Failure Guidelines for the Management of Heart Failure: Management of the Underlying Etiologies and Comorbidities of Heart Failure	None recommendation of any prediction model
Database	Japan Atherosclerosis Society (JAS) Guidelines for Prevention of Atherosclerotic Cardiovascular Diseases 2022	No explicit recommendation of specific prediction model
Database	American Society of Hematology, ABHH, ACHO, Grupo CAHT, Grupo CLAHT, SAH, SBHH, SHU, SOCHIHEM, SOMETH, Sociedad Panamena de Hematología, Sociedad Peruana de Hematología, and SVH 2023 guidelines for diagnosis of venous thromboembolism and for its management in special populations in Latin America	None recommendation of any prediction model
Database	British Society of Echocardiography guideline for the transthoracic echocardiographic assessment of cardiac amyloidosis	None recommendation of any prediction model
Database	2023 Focused Update of the 2021 ESC Guidelines for the diagnosis and treatment of acute and chronic heart failure Developed by the task force for the diagnosis and treatment of acute and chronic heart failure of the European Society of Cardiology (ESC) With the special contribution of the Heart Failure Association (HFA) of the ESC	None recommendation of any prediction model
Database	French guidelines for the treatment of cancer-associated venous thromboembolism - 2023 update	None recommendation of any prediction model
Database	2023 Chinese guideline for lipid management	None recommendation of any prediction model
Database	2023 American Heart Association Focused Update on the Management of Patients With Cardiac Arrest or Life-Threatening Toxicity Due to Poisoning:	None recommendation of any prediction model

	An Update to the American Heart Association Guidelines for Cardiopulmonary Resuscitation and Emergency Cardiovascular Care	
Database	ACR Appropriateness Criteria® Congenital or Acquired Heart Disease	Inconformity with definition of CPG
Database	PEER simplified lipid guideline 2023 update: Prevention and management of cardiovascular disease in primary care	No explicit recommendation of specific prediction model
Database	The 2023 Canadian Cardiovascular Society Clinical Practice Update on Management of the Patient With a Prolonged QT Interval	Inconformity with definition of CPG
Database	2023 HFCT Focused Update of the 2019 HFCT Heart Failure Guidelines Part 2: Diagnosis and Management of HFmrEF and HFpEF	None recommendation of any prediction model
Database	2020 ESC Guidelines for the management of acute coronary syndromes in patients presenting without persistent ST-segment elevation	Older version
Database	Focused Update on Patients Treated with the Nellix EndoVascular Aneurysm Sealing (EVAS) System from the European Society for Vascular Surgery (ESVS) Abdominal Aortic Aneurysm Clinical Practice Guidelines	None recommendation of any prediction model
Database	The International Society for Heart and Lung Transplantation/Heart Failure Society of America Guideline on Acute Mechanical Circulatory Support	None recommendation of any prediction model
Database	Updated guidelines for the management of dyslipidemia and the prevention of cardiovascular disease in adults by pharmacists	Inconformity with definition of CPG
Database	New-onset atrial fibrillation in critically ill adult patients-an SSAI clinical practice guideline	None recommendation of any prediction model
Database	National Heart Center/Saudi Heart Association 2023 Guidelines on the Management of Hypertension	No explicit recommendation of specific prediction model
Database	2023 National Heart Center/Saudi Heart Association Focused Update of the 2019 Saudi Heart Association Guidelines for the Management of Heart Failure	No explicit recommendation of specific prediction model
Database	Interdisciplinary German clinical practice guidelines on the management of type B aortic dissection	Inconformity with definition of CPG
Database	Clinical practice guideline for the prevention and management of hypertensive disorders of pregnancy	Non-English text
Database	Australian and New Zealand Society for Vascular Surgery clinical practice guidelines on venous outflow Obstruction of the femoral-iliocaval veins	Withdrawn publication
Database	2022 Guidelines of the Taiwan Society of Cardiology and the Taiwan Hypertension Society for the Management of Hypertension	None recommendation of any prediction model
Database	2022 Vietnamese Society of Hypertension guidelines for the diagnosis and treatment of arterial hypertension: The collaboration of the Vietnamese Society of Hypertension (VSH) task force with the contribution of the Vietnam National Heart Association (VNHA)	None recommendation of any prediction model
Database	2021 ESC/EACTS Guidelines for the management of valvular heart disease	None recommendation of any prediction model
Database	Guideline for Venous ThromboEmbolism(VTE) Prophylaxis for patients admitted to hospice	Inconformity with definition of CPG

Database	Guideline No. 417: Prevention of Venous Thromboembolic Disease in Gynaecological Surgery	None recommendation of any prediction model
Database	ACR Appropriateness Criteria® Suspected Pulmonary Hypertension: 2022 Update	Inconformity with definition of CPG
Database	Guidelines for physical activity in children with heart disease	None recommendation of any prediction model
Database	ISTH guidelines for antithrombotic treatment in COVID-19	None recommendation of any prediction model
Database	European Stroke Organisation (ESO) guideline on screening for subclinical atrial fibrillation after stroke or transient ischaemic attack of undetermined origin	None recommendation of any prediction model
Database	Guidelines on the management of abdominal aortic aneurysms: Updates from the Italian Society of Vascular and Endovascular Surgery (SICVE)	None recommendation of any prediction model
Database	JCS 2022 Guideline on Management and Re-Interventional Therapy in Patients With Congenital Heart Disease Long-Term After Initial Repair	None recommendation of any prediction model
Database	Canadian Society of Thoracic Radiology/Canadian Association of Radiologists Best Practice Guidance for Investigation of Acute Pulmonary Embolism, Part 1: Acquisition and Safety Considerations	Inconformity with definition of CPG
Database	Canadian Society of Thoracic Radiology/Canadian Association of Radiologists Best Practice Guidance for Investigation of Acute Pulmonary Embolism, Part 2: Technical Issues and Interpretation Pitfalls	Inconformity with definition of CPG
Database	JCS 2022 Guideline Focused Update on Diagnosis and Treatment in Patients With Stable Coronary Artery Disease	None recommendation of any prediction model
Database	Canadian Cardiovascular Society 2022 Guidelines for Cardiovascular Interventions in Adults With Congenital Heart Disease	None recommendation of any prediction model
Database	2022 Canadian Cardiovascular Society Guideline for Use of GLP-1 Receptor Agonists and SGLT2 Inhibitors for Cardiorenal Risk Reduction in Adults	None recommendation of any prediction model
Database	Guideline No. 426: Hypertensive Disorders of Pregnancy: Diagnosis, Prediction, Prevention, and Management	None recommendation of any prediction model
Database	The 2021 International Society for the Study of Hypertension in Pregnancy classification, diagnosis & management recommendations for international practice	No explicit recommendation of specific prediction model
Database	The Society of Thoracic Surgeons/American Association for Thoracic Surgery clinical practice guidelines on the management of type B aortic dissection	None recommendation of any prediction model
Database	Guidelines for Echocardiographic Diagnosis of Cardiomyopathy: Recommendations from Echocardiography Group of Ultrasound Medicine Branch in Chinese Medical Association, Echocardiography Committee of Cardiovascular Branch in Chinese Medical Association	None recommendation of any prediction model
Database	ACR Appropriateness Criteria® Chronic Chest Pain-High Probability of Coronary Artery Disease: 2021 Update	Inconformity with definition of CPG
Database	Clinical Practice Guidelines in Cardio-Oncology	Inconformity with definition

		of CPG
Database	RSSDI Guidelines for the management of hypertension in patients with diabetes mellitus	None recommendation of any prediction model
Database	The Detection, Evaluation, and Management of Dyslipidemia in Children and Adolescents: A Canadian Cardiovascular Society/Canadian Pediatric Cardiology Association Clinical Practice Update	None recommendation of any prediction model
Database	2022 Taiwan lipid guidelines for primary prevention	No explicit recommendation of specific prediction model
Database	PCSK9 inhibitors and ezetimibe for the reduction of cardiovascular events: a clinical practice guideline with risk-stratified recommendations	No explicit recommendation of specific prediction model
Database	2022 ESC Guidelines on cardiovascular assessment and management of patients undergoing non-cardiac surgery	No explicit recommendation of specific prediction model
Database	2021 AHA/ACC/ASE/CHEST/SAEM/SCCT/SCMR Guideline for the Evaluation and Diagnosis of Chest Pain	None recommendation of any prediction model
Database	2022 Practice guidelines for the management of arterial hypertension of the Spanish Society of Hypertension	Non-English text
Database	2021 ESC Guidelines on cardiac pacing and cardiac resynchronization therapy	None recommendation of any prediction model
Database	The Society for Vascular Surgery clinical practice guidelines on popliteal artery aneurysms	None recommendation of any prediction model
Database	Joint British Societies' guideline on management of cardiac arrest in the cardiac catheter laboratory	None recommendation of any prediction model
Database	Perioperative Management of Antithrombotic Therapy: An American College of Chest Physicians Clinical Practice Guideline	No explicit recommendation of specific prediction model
Database	Clinical practice guideline of the Interamerican Society of Cardiology on primary prevention of cardiovascular disease in women	Non-English text
Database	Update of the Brazilian Society of Cardiology's Perioperative Cardiovascular Assessment Guideline: Focus on Managing Patients with Percutaneous Coronary Intervention – 2022	None recommendation of any prediction model
Database	Evidence-Based Guidelines for Acute Stabilization and Management of Neonates with Persistent Pulmonary Hypertension of the Newborn	Inconformity with definition of CPG
Database	Use of extracorporeal circulation (ECLS/ECMO) for cardiac and circulatory failure -A clinical practice Guideline Level 3	None recommendation of any prediction model
Database	2022 HFCT Focused Update of the 2019 HFCT Heart Failure Guidelines: Part 1 - Heart Failure Classification and Pharmacological Treatment for Heart Failure with Reduced Ejection Fraction (HFrEF)	None recommendation of any prediction model
Database	Thrombophilia testing: A British Society for Haematology guideline	None recommendation of any prediction model
Database	Clinical practice guideline for the prevention and management of hypertensive disorders of pregnancy	Non-English text
Database	JCS 2018 Guideline on Diagnosis of Chronic Coronary Heart Diseases	No explicit recommendation of specific prediction model

Database	Canadian Cardiovascular Society: Clinical Practice Update on Cardiovascular Management of the Pregnant Patient	Inconformity with definition of CPG
Database	Hypertension Canada’s 2020 hypertension guidelines for pharmacists: An update	None recommendation of any prediction model
Database	2021 ESC/EACTS Guidelines for the management of valvular heart disease	None recommendation of any prediction model
Database	Transthoracic echocardiography of hypertrophic cardiomyopathy in adults: A practical guideline from the British Society of Echocardiography	None recommendation of any prediction model
Database	Guideline Update on Indications for Transcatheter Aortic Valve Implantation Based on the 2020 American College of Cardiology/American Heart Association Guidelines for Management of Valvular Heart Disease	Inconformity with definition of CPG
Database	Antithrombotic Therapy for VTE Disease: Second Update of the CHEST Guideline and Expert Panel Report	None recommendation of any prediction model
Database	2021 European Society of Hypertension practice guidelines for office and out-of-office blood pressure measurement	Inconformity with definition of CPG
Database	European Resuscitation Council Guidelines 2021: Systems saving lives	Inconformity with definition of CPG
Database	Joint Trauma System Clinical Practice Guideline: Acute Coronary Syndrome (ACS). 14 May 2021	Inconformity with definition of CPG
Database	ESC/EACTS 2021 Guidelines on the treatment of valvulopathies	Non-English text
Database	The 2020 Australian guideline for prevention, diagnosis and management of acute rheumatic fever and rheumatic heart disease	No explicit recommendation of specific prediction model
Database	ACR Appropriateness Criteria® Nonischemic Myocardial Disease with Clinical Manifestations (Ischemic Cardiomyopathy Already Excluded)	Inconformity with definition of CPG
Database	The German-Austrian S3 Guideline Cardiogenic Shock Due to Myocardial Infarction: Diagnosis, Monitoring, and Treatment	None recommendation of any prediction model
Database	2020 ESC Guidelines on sports cardiology and exercise in patients with cardiovascular disease	No explicit recommendation of specific prediction model
Database	2021 Canadian Cardiovascular Society Guidelines for the Management of Dyslipidemia for the Prevention of Cardiovascular Disease in Adults	No explicit recommendation of specific prediction model
Database	2020 AHA/ACC guideline for the diagnosis and treatment of patients with hypertrophic cardiomyopathy	No explicit recommendation of specific prediction model
Database	JCS/JHRS 2019 Guideline on Non-Pharmacotherapy of Cardiac Arrhythmias	None recommendation of any prediction model
Database	CCS/CHFS Heart Failure Guidelines Update: Defining a New Pharmacologic Standard of Care for Heart Failure With Reduced Ejection Fraction	None recommendation of any prediction model
Database	2021 ESC Guidelines for the diagnosis and treatment of acute and chronic heart failure	No explicit recommendation of specific prediction model
Database	ACR Appropriateness Criteria® Infective Endocarditis	Inconformity with definition of CPG
Database	European Resuscitation Council Guidelines 2021: Cardiac arrest in special circumstances	Inconformity with definition of CPG

Database	SGLT-2 inhibitors or GLP-1 receptor agonists for adults with type 2 diabetes: a clinical practice guideline	None recommendation of any prediction model
Database	Clinical practice guideline for transcatheter versus surgical valve replacement in patients with severe aortic stenosis in Latin America	None recommendation of any prediction model
Database	Guidelines on the prevention and treatment of venous thromboembolism in cancer patients treated surgically, including patients under 18 years of age	No explicit recommendation of specific prediction model
Database	The 2020 ESC guidelines on the diagnosis and management of atrial fibrillation	Inconformity with definition of CPG
Database	2020 Korean guidelines for cardiopulmonary resuscitation. part 5. post-cardiac arrest care	None recommendation of any prediction model
Database	ACR Appropriateness Criteria® Nontraumatic Aortic Disease	Inconformity with definition of CPG
Database	ACR Appropriateness Criteria® Asymptomatic Patient at Risk for Coronary Artery Disease: 2021 Update	Inconformity with definition of CPG
Database	Guidelines for screening and managing hypertension in children	None recommendation of any prediction model
Database	Italian Cardiological Guidelines (COCIS) for Competitive Sport Eligibility in athletes with heart disease: update 2020	Inconformity with definition of CPG
Database	Singapore Advanced Cardiac Life Support Guidelines 2021	None recommendation of any prediction model
Database	2021 Focused update of the 2017 consensus guidelines of the Asia Pacific Heart Rhythm Society (APHRS) on stroke prevention in atrial fibrillation	Inconformity with definition of CPG
Database	Extracorporeal Life Support Organization (ELSO): Guidelines for Pediatric Cardiac Failure	None recommendation of any prediction model
Database	Guidelines for the management of women with severe pre-eclampsia	None recommendation of any prediction model
Database	2020 ESC Guidelines for the management of adult congenital heart disease	None recommendation of any prediction model
Database	Part 5: Neonatal Resuscitation 2020 American Heart Association Guidelines for Cardiopulmonary Resuscitation and Emergency Cardiovascular Care	None recommendation of any prediction model
Database	Guideline No. 422e: Menopause and Cardiovascular Disease	None recommendation of any prediction model
Database	Clinical Practice Guidelines. Management of Hypertension in Tunisian Adults	Non-English text
Database	Interinstitutional clinical practice guidelines for the treatment of acute myocardial infarction	Inconformity with definition of CPG
Database	National Clinical Guideline: Atherosclerotic Cardiovascular Disease Risk Assessment and Management (2020)	No explicit recommendation of specific prediction model
Database	2019 Chinese Hypertension League guidelines on home blood pressure monitoring	None recommendation of any prediction model
Database	2019 EACTS/EACTA/EBCP guidelines on cardiopulmonary bypass in adult cardiac surgery	None recommendation of any prediction model
Database	Guidelines for the Treatment of Pulmonary Arterial Hypertension	Inconformity with definition

		of CPG
Database	2020 International Society of Hypertension Global Hypertension Practice Guidelines	None recommendation of any prediction model
Database	National Clinical Guidance for the Management of Cardiovascular Intervention in the COVID-19 Pandemic: From Bangladesh Society of Cardiovascular Interventions (BSCI)	None recommendation of any prediction model
Database	Part 4: Pediatric Basic and Advanced Life Support: 2020 American Heart Association Guidelines for Cardiopulmonary Resuscitation and Emergency Cardiovascular Care	None recommendation of any prediction model
Database	Physical Therapist Clinical Practice Guideline for the Management of Individuals With Heart Failure	None recommendation of any prediction model
Database	Indian guidelines on hypertension-IV (2019)	None recommendation of any prediction model
Database	Hypertension Canada's 2020 Comprehensive Guidelines for the Prevention, Diagnosis, Risk Assessment, and Treatment of Hypertension in Adults and Children	No explicit recommendation of specific prediction model
Database	Guidelines for Performance, Interpretation, and Application of Stress Echocardiography in Ischemic Heart Disease: From the American Society of Echocardiography	None recommendation of any prediction model
Database	Part 3: Adult Basic and Advanced Life Support: 2020 American Heart Association Guidelines for Cardiopulmonary Resuscitation and Emergency Cardiovascular Care	None recommendation of any prediction model
Database	American Society of Hematology 2020 guidelines for management of venous thromboembolism: treatment of deep vein thrombosis and pulmonary embolism	No explicit recommendation of specific prediction model
Database	Diagnosing acute aortic syndrome: a Canadian clinical practice guideline	No explicit recommendation of specific prediction model
Database	CCS/CHFS Heart Failure Guidelines: Clinical Trial Update on Functional Mitral Regurgitation, SGLT2 Inhibitors, ARNI in HFpEF, and Tafamidis in Amyloidosis	None recommendation of any prediction model
Database	Management of Dyslipidemia for Cardiovascular Disease Risk Reduction: Synopsis of the 2020 Updated U.S. Department of Veterans Affairs and U.S. Department of Defense Clinical Practice Guideline	No explicit recommendation of specific prediction model
Database	JCS 2020 Guideline Focused Update on Antithrombotic Therapy in Patients With Coronary Artery Disease	None recommendation of any prediction model
Database	Prevention, Diagnosis, and Treatment of VTE in Patients With Coronavirus Disease 2019: CHEST Guideline and Expert Panel Report	None recommendation of any prediction model
Database	Update of the Brazilian Guideline on Nuclear Cardiology - 2020	No explicit recommendation of specific prediction model
Database	Consensus Guidelines for International Cardiology Services Delivery During COVID-19 Pandemic in Australia and New Zealand	Inconformity with definition of CPG
Database	2017 Clinical practice guidelines for dyslipidemia of Korean children and	None recommendation of any

	adolescents	prediction model
Database	2020 focused update of the 2012 guidelines of the taiwan society of cardiology for the management of st-segment elevation myocardial infarction	None recommendation of any prediction model
Database	2019 ESC Guidelines for the diagnosis and management of acute pulmonary embolism developed in collaboration with the European respiratory society (ERS)	None recommendation of any prediction model
Database	Society for Vascular Nursing endovascular repair of abdominal aortic aneurysm updated nursing clinical practice guideline	None recommendation of any prediction model
Database	JCS 2020 Guideline on Diagnosis and Treatment of Cardiac Amyloidosis	None recommendation of any prediction model
Database	American Association for Thoracic Surgery/International Society for Heart and Lung Transplantation guidelines on selected topics in mechanical circulatory support	Inconformity with definition of CPG
Database	Society of Interventional Radiology Clinical Practice Guideline for Inferior Vena Cava Filters in the Treatment of Patients with Venous Thromboembolic Disease	None recommendation of any prediction model
Database	JCS/JSCS/JATS/JSVS 2020 Guidelines on the Management of Valvular Heart Disease	None recommendation of any prediction model
Database	Hypertension Canada's 2020 Evidence Review and Guidelines for the Management of Resistant Hypertension	None recommendation of any prediction model
Database	2019 American Heart Association Focused Update on Pediatric Advanced Life Support: An Update to the American Heart Association Guidelines for Cardiopulmonary Resuscitation and Emergency Cardiovascular Care	Older version
Database	Update: AHA guidelines for CPR and emergency cardiovascular care	None recommendation of any prediction model
Database	2019 ESC Guidelines on diabetes, pre-diabetes, and cardiovascular diseases developed in collaboration with the EASD	No explicit recommendation of specific prediction model
Database	The Society for Vascular Surgery clinical practice guidelines on the management of visceral aneurysms	None recommendation of any prediction model
Database	2019 ESC Guidelines for themanagement of patients with supraventricular tachycardia	None recommendation of any prediction model
Database	Part 7: Systems of care 2020 american heart association guidelines for cardiopulmonary resuscitation and emergency cardiovascular care	None recommendation of any prediction model
Database	Pediatric hypertension: A guideline update	None recommendation of any prediction model
Database	The ESC Clinical Practice Guidelines for the Management of Adult Congenital Heart Disease 2020	Inconformity with definition of CPG
Database	ACR Appropriateness Criteria® Chest Pain-Possible Acute Coronary Syndrome	Inconformity with definition of CPG
Database	Part 5: Neonatal Resuscitation: 2020 American Heart Association Guidelines for Cardiopulmonary Resuscitation and Emergency Cardiovascular Care	None recommendation of any prediction model
Database	The 2020 Canadian Cardiovascular Society/Canadian Heart Rhythm Society	No explicit recommendation

	Comprehensive Guidelines for the Management of Atrial Fibrillation	of specific prediction model
Database	Guidelines for the management of neonates and infants with hypoplastic left heart syndrome	None recommendation of any prediction model
Database	Guidelines for the Evaluation of Valvular Regurgitation After Percutaneous Valve Repair or Replacement	None recommendation of any prediction model
Database	2019 Canadian Cardiovascular Society/Canadian Association of Interventional Cardiology Guidelines on the Acute Management of ST-Elevation Myocardial Infarction: Focused Update on Regionalization and Reperfusion	None recommendation of any prediction model
Database	European Society for Vascular Surgery (ESVS) 2019 Clinical Practice Guidelines on the Management of Abdominal Aorto-iliac Artery Aneurysms	Older version
Database	2019 focused update of the guidelines of the Taiwan society of cardiology for the diagnosis and treatment of heart failure	No explicit recommendation of specific prediction model
Database	The Japanese Society of Hypertension Guidelines for the Management of Hypertension (JSH 2019)	No explicit recommendation of specific prediction model
Database	S2k guidelines for the diagnosis and treatment of type B aortic dissection	Inconformity with definition of CPG
Database	2019 international clinical practice guidelines for the treatment of venous thromboembolism	Inconformity with definition of CPG
Database	2018 AHA/ACC Guideline for the Management of Adults With Congenital Heart Disease	None recommendation of any prediction model
Database	Stress echocardiography in coronary artery disease: A practical guideline from the British Society of Echocardiography	Inconformity with definition of CPG
Database	European Resuscitation Council Guidelines for Resuscitation: 2018 Update - Antiarrhythmic drugs for cardiac arrest	None recommendation of any prediction model
Database	ESC Guidelines for the management of cardiovascular diseases during pregnancy (2018)	No explicit recommendation of specific prediction model
Database	Heart failure council of Thailand (HFCT) 2019 heart failure guideline: Acute heart failure	None recommendation of any prediction model
Database	Heart failure council of Thailand (HFCT) 2019 heart failure guideline: Comorbidity in heart failure	None recommendation of any prediction model
Database	Heart failure council of Thailand (HFCT) 2019 heart failure guideline: Sudden cardiac death and device therapy in heart failure	None recommendation of any prediction model
Database	Heart failure council of thailand (HFCT) 2019 heart failure guideline: Pharmacologic treatment of chronic heart failure - Part I	Older version
Database	Heart Failure Council of Thailand (HFCT) 2019 Heart Failure Guideline: Pharmacologic Treatment of Chronic Heart Failure - Part II	None recommendation of any prediction model
Database	Heart failure council of Thailand (HFCT) 2019 heart failure guideline: Introduction and diagnosis	None recommendation of any prediction model
Database	Heart Failure Council of Thailand (HFCT) 2019 Heart Failure Guideline: Advanced Heart Failure	None recommendation of any prediction model
Database	Brazilian fetal cardiology guidelines – 2019	Older version

Database	Society of Interventional Radiology Consensus Guidelines for the Periprocedural Management of Thrombotic and Bleeding Risk in Patients Undergoing Percutaneous Image-Guided Interventions-Part II	No explicit recommendation of specific prediction model
Database	Guidelines for Performing a Comprehensive Transthoracic Echocardiographic Examination in Adults: Recommendations from the American Society of Echocardiography	None recommendation of any prediction model
Database	French Society of Cardiology guidelines on exercise tests (part 2): Indications for exercise tests in cardiac diseases	None recommendation of any prediction model
Database	Guideline of the Brazilian Society of Cardiology on Telemedicine in Cardiology - 2019	None recommendation of any prediction model
Database	KSHF guidelines for the management of acute heart failure: Part I. Definition, epidemiology and diagnosis of acute heart failure	No explicit recommendation of specific prediction model
Database	2018 ACC/AHA/HRS guideline on the evaluation and management of patients with bradycardia and cardiac conduction delay	No explicit recommendation of specific prediction model
Database	KSHF guidelines for the management of acute heart failure: Part III. Specific Management of Acute Heart Failure According to the Etiology and Co-morbidity	None recommendation of any prediction model
Database	Clinical Practice Guideline for Cardiac Rehabilitation in Korea	None recommendation of any prediction model
Database	2018 TSOC guideline focused update on diagnosis and treatment of pulmonary arterial hypertension	None recommendation of any prediction model
Database	Guidelines for the Treatment of Pulmonary Hypertension (JCS 2017/JPCPHS 2017)	No explicit recommendation of specific prediction model
Database	ESVM Guideline on peripheral arterial disease	No explicit recommendation of specific prediction model
Database	Saudi Heart Association (SHA) guidelines for the management of heart failure (2019)	Older version
Database	2018 ESC/ESH Guidelines for themanagement of arterial hypertension	Older version
Database	2017 ACC/AHA/AAPA/ABC/ACPM/AGS/APhA/ASH/ASPC/NMA/PCNA Guideline for the Prevention, Detection, Evaluation, and Management of High Blood Pressure in Adults	None recommendation of any prediction model
Database	Canadian Cardiovascular Harmonized National Guidelines Endeavour (C-CHANGE) guideline for the prevention and management of cardiovascular disease in primary care: 2018 update	Older version
Database	2018 American Heart Association Focused Update on Advanced Cardiovascular Life Support Use of Antiarrhythmic Drugs During and Immediately After Cardiac Arrest	None recommendation of any prediction model
Database	Management of Atherosclerotic Carotid and Vertebral Artery Disease: 2017 Clinical Practice Guidelines of the European Society for Vascular Surgery (ESVS)	Older version
Database	American Society of Hematology 2018 Guidelines for management of venous thromboembolism: treatment of pediatric venous thromboembolism	None recommendation of any prediction model

Database	2018 Canadian Cardiovascular Society/Canadian Association of Interventional Cardiology Focused Update of the Guidelines for the Use of Antiplatelet Therapy	No explicit recommendation of specific prediction model
Database	Guidelines of the pediatric section of the polish society of hypertension on diagnosis and treatment of arterial hypertension in children and adolescents	None recommendation of any prediction model
Database	Antithrombotic Therapy for Atrial Fibrillation: CHEST Guideline and Expert Panel Report	Older version
Database	American Society of Hematology 2018 guidelines for management of venous thromboembolism: diagnosis of venous thromboembolism	No explicit recommendation of specific prediction model
Database	2017 Update of ESC/EAS Task Force on practical clinical guidance for proprotein convertase subtilisin/kexin type 9 inhibition in patients with atherosclerotic cardiovascular disease or in familial hypercholesterolaemia	None recommendation of any prediction model
Database	Hypertension Canada’s 2017 guidelines for diagnosis, risk assessment, prevention and treatment of hypertension in adults for pharmacists: An update	Older version
Database	2017 American Heart Association Focused Update on Adult Basic Life Support and Cardiopulmonary Resuscitation Quality: An Update to the American Heart Association Guidelines for Cardiopulmonary Resuscitation and Emergency Cardiovascular Care	Older version
Database	Japan Atherosclerosis Society (JAS) Guidelines for Prevention of Atherosclerotic Cardiovascular Diseases 2017	Older version
Database	2017 ESC Guidelines for the management of acute myocardial infarction in patients presenting with ST-segment elevation	No explicit recommendation of specific prediction model
Database	2017 ESC Guidelines on the Diagnosis and Treatment of Peripheral Arterial Diseases	No explicit recommendation of specific prediction model
Database	2018 American Heart Association Focused Update on Pediatric Advanced Life Support: An Update to the American Heart Association Guidelines for Cardiopulmonary Resuscitation and Emergency Cardiovascular Care	Older version
Database	Hypertension Canada's 2018 Guidelines for the Management of Hypertension in Pregnancy	None recommendation of any prediction model
Database	American Society of Hematology 2018 guidelines for management of venous thromboembolism: Venous thromboembolism in the context of pregnancy	None recommendation of any prediction model
Database	2017 American Heart Association Focused Update on Pediatric Basic Life Support and Cardiopulmonary Resuscitation Quality: An Update to the American Heart Association Guidelines for Cardiopulmonary Resuscitation and Emergency Cardiovascular Care	Older version
Database	National Heart Foundation of Australia and Cardiac Society of Australia and New Zealand: Guidelines for the Prevention, Detection, and Management of Heart Failure in Australia 2018	None recommendation of any prediction model
Database	2018 Focused Update of the Canadian Cardiovascular Society Guidelines for the Management of Atrial Fibrillation	Older version
Database	2017 AHA/ACC/HRS guideline for management of patients with ventricular arrhythmias and the prevention of sudden cardiac death	None recommendation of any prediction model

Database	European guidelines on perioperative venous thromboembolism prophylaxis	Inconformity with definition of CPG
WHO	WHO recommendations on drug treatment for non-severe hypertension in pregnancy	None recommendation of any prediction model
WHO	WHO recommendations: drug treatment for severe hypertension in pregnancy	None recommendation of any prediction model
NICE	Peripheral arterial disease: diagnosis and management (CG147)	None recommendation of any prediction model
NICE	Acute heart failure: diagnosis and management (CG187)	None recommendation of any prediction model
NICE	Subarachnoid haemorrhage caused by a ruptured aneurysm: diagnosis and management (NG228)	No explicit recommendation of specific prediction model
NICE	Venous thromboembolism in over 16s: reducing the risk of hospital-acquired deep vein thrombosis or pulmonary embolism (NG89)	No explicit recommendation of specific prediction model
NICE	Chronic heart failure in adults: diagnosis and management (NG106)	None recommendation of any prediction model
NICE	Hypertension in pregnancy: diagnosis and management (NG133)	None recommendation of any prediction model
NICE	Hypertension in adults: diagnosis and management (NG136)	None recommendation of any prediction model
NICE	COVID-19 rapid guideline: vaccine-induced immune thrombocytopenia and thrombosis (VITT) (NG200)	None recommendation of any prediction model
NICE	Heart valve disease presenting in adults: investigation and management (NG208)	None recommendation of any prediction model
SIGN	Management of stable angina (SIGN151)	None recommendation of any prediction model
SIGN	Cardiac arrhythmias in coronary heart disease (SIGN152)	None recommendation of any prediction model
GIN	American Society of Hematology 2020 guidelines for management of venous thromboembolism: treatment of deep vein thrombosis and pulmonary embolism	No explicit recommendation of specific prediction model
GIN	National Heart Foundation of Australia and Cardiac Society of Australia and New Zealand: Guidelines for the prevention, detection and management of heart failure in Australia 2018	None recommendation of any prediction model
GIN	Women-Focused Cardiovascular Rehabilitation: An International Council of Cardiovascular Prevention and Rehabilitation Clinical Practice Guideline	None recommendation of any prediction model
GIN	Depression Following Acute Coronary Syndrome Events: Screening and Treatment Guidelines from the AAFP	No explicit recommendation of specific prediction model
CMACPG	The detection, evaluation, and management of Dyslipidemia in children and adolescents: a Canadian Cardiovascular Society/Canadian Pediatric Cardiology Association clinical practice update	None recommendation of any prediction model
CMACPG	2021 Update on safety of magnetic resonance imaging: joint statement from	None recommendation of any

	Canadian Cardiovascular Society/Canadian Society for Cardiovascular Magnetic Resonance/Canadian Heart Rhythm Society	prediction model
CMACPG	Canadian Cardiovascular Society: clinical practice update on cardiovascular management of the pregnant patient	No explicit recommendation of specific prediction model
CMACPG	EACVI recommendations on cardiovascular imaging for the detection of embolic sources: endorsed by the Canadian Society of Echocardiography	None recommendation of any prediction model
CMACPG	Guidance on community-based care of the cardiovascular patient during the COVID-19 pandemic	None recommendation of any prediction model
CMACPG	Ramping up the delivery of cardiac surgery during the COVID-19 pandemic: a guidance statement from the Canadian Society of Cardiac Surgeons	None recommendation of any prediction model
CMACPG	Cardiac surgery in Canada during the COVID-19 pandemic: a guidance statement from the Canadian Society of Cardiac Surgeons	None recommendation of any prediction model
CMACPG	The New "virtual reality": practical approaches to the delivery of cardiac rehabilitation care during the COVID-19 crisis	No explicit recommendation of specific prediction model
CMACPG	Canadian Cardiovascular Society/Canadian Cardiac Transplant Network position statement on heart transplantation: patient eligibility, selection, and post-transplantation Care	None recommendation of any prediction model

Supplementary Table 5 Characteristic of Included CPGs

Guideline ID	Developed/ Updated year	Name	Location	Country	Organization(s)	Criteria for forming recommendations
Virani 2023	2023	2023 AHA/ACC/ACCP/ASPC/NLA/PCNA Guideline for the Management of Patients With Chronic Coronary Disease: A Report of the American Heart Association/American College of Cardiology Joint Committee on Clinical Practice Guidelines	North America	USA	AHA/ACC/ACCP/ASPC/NLA/PCNA	ACCF/AHA approach
Ueng 2023	2023	2023 Guidelines of the Taiwan Society of Cardiology on the Diagnosis and Management of Chronic Coronary Syndrome	Asia	China	TSC	ESC approach
Takase 2023	2023	JCS/JHRS 2022 Guideline on Diagnosis and Risk Assessment of Arrhythmia	Asia	Japan	JCS/JHRS	ACCF/AHA approach
Rajajee 2023	2023	Guidelines for Neuroprognostication in Comatose Adult Survivors of Cardiac Arrest	Europe	Germany	NCS/DGN	GRADE approach
Key 2023	2023	Venous Thromboembolism Prophylaxis and Treatment in Patients With Cancer: ASCO Guideline Update	North America	USA	ASCO	modified GRADE approach
Hiraoka 2023	2023	JCS 2022 Guideline on Perioperative Cardiovascular Assessment and Management for Non-Cardiac Surgery	Asia	Japan	JCS	ACCF/AHA approach
Falanga 2023	2023	Venous thromboembolism in cancer patients: ESMO Clinical Practice Guideline	Europe	/	ESMO	ESMO approach
Byrne 2023	2023	2023 ESC Guidelines for the management of acute coronary syndromes	Europe	/	ESC	ESC approach
Arbelo 2023	2023	2023 ESC Guidelines for the management of cardiomyopathies	Europe	/	ESC	ESC approach
NG238	2023	Cardiovascular disease: risk assessment and reduction, including lipid modification	Europe	UK	NICE	modified GRADE approach
NG158	2023	Venous thromboembolic diseases: diagnosis, management and thrombophilia testing	Europe	UK	NICE	modified GRADE approach
Zeppenfeld 2022	2022	2022 ESC Guidelines for the management of patients with ventricular arrhythmias and the prevention of sudden cardiac death	Europe	/	ESC	ESC approach
Lyon 2022	2022	2022 ESC Guidelines on cardio-oncology developed in collaboration with the European Hematology Association (EHA), the European Society for Therapeutic Radiology and Oncology (ESTRO) and the International Cardio-Oncology Society (IC-OS)	Europe	/	ESC/EHA/ESTRO/IC-OS	ESC approach

Jain 2022	2022	Canadian Cardiovascular Harmonized National Guideline Endeavour (C-CHANGE) guideline for the prevention and management of cardiovascular disease in primary care: 2022 update	North America	Canada	C-CHANGE	modified GRADE approach
Hillegass 2022	2022	Role of Physical Therapists in the Management of Individuals at Risk for or Diagnosed With Venous Thromboembolism: Evidence-Based Clinical Practice Guideline 2022	North America	USA	APTH	APTH approach
Heidenreich 2022	2022	2022 AHA/ACC/HFSA Guideline for the Management of Heart Failure: A Report of the American College of Cardiology/American Heart Association Joint Committee on Clinical Practice Guidelines	North America	USA	ACC/AHA/HFSA	ACCF/AHA approach
Farge 2022	2022	2022 international clinical practice guidelines for the treatment and prophylaxis of venous thromboembolism in patients with cancer, including patients with COVID-19	Worldwide	/	ITAC	GRADE approach
Albricker 2022	2022	Joint Guideline on Venous Thromboembolism, 2022	South America	Brazil	BSC/BCR/BSAVS/BSNM	modified GRADE approach
Visseren 2021	2021	2021 ESC Guidelines on cardiovascular disease prevention in clinical practice	Europe	/	ESC	ESC approach
Tsutsui 2021	2021	JCS/JHFS 2021 Guideline Focused Update on Diagnosis and Treatment of Acute and Chronic Heart Failure	Asia	Japan	JCS/JHFS	ACCF/AHA approach
Lawton 2021	2021	2021 ACC/AHA/SCAI Guideline for Coronary Artery Revascularization: A Report of the American College of Cardiology/American Heart Association Joint Committee on Clinical Practice Guidelines	North America	USA	ACC/AHA/SCAI/STS	ACCF/AHA approach
Kakkos 2021	2021	European Society for Vascular Surgery (ESVS) 2021 Clinical Practice Guidelines on the Management of Venous Thrombosis	Europe	/	ESVS	ESC approach
NG196	2021	Atrial fibrillation: diagnosis and management	Europe	UK	NICE	modified GRADE approach
BCG 2021	2021	Cardiovascular Disease – Primary Prevention	North America	Canada	GPAC	modified GRADE approach
Otto 2021	2020	2020 ACC/AHA Guideline for the Management of Patients With Valvular Heart Disease: A Report of the American College of Cardiology/American Heart Association Joint Committee on Clinical Practice Guidelines	North America	USA	ACC/AHA	ACCF/AHA approach

Hindricks 2021	2020	2020 ESC Guidelines for the diagnosis and management of atrial fibrillation developed in collaboration with the European Association for Cardio-Thoracic Surgery (EACTS)	Europe	/	ESC/EACTS/EHRA	ESC approach
Liao 2020	2020	Clinical Practice Guideline of Integrative Chinese and Western Medicine for Acute Myocardial Infarction	Asia	China	CMDA/CAIM/CACM	GRADE approach
Hajjar 2020	2020	Brazilian cardio-oncology guideline-2020	South America	Brazil	BSC/BSCO	ESC approach
NG156	2020	Abdominal aortic aneurysm: diagnosis and management	Europe	UK	NICE	modified GRADE approach
NG185	2020	Acute coronary syndromes	Europe	UK	NICE	modified GRADE approach
Newman 2020	2020	Lipid Management in Patients with Endocrine Disorders: An Endocrine Society Clinical Practice Guideline	North America	USA	AACE/ESE	modified GRADE approach
Mach 2020	2019	2019 ESC/EAS Guidelines for the management of dyslipidaemias: Lipid modification to reduce cardiovascular risk	Europe	/	ESC/EAS	ESC approach
Knuuti 2020	2019	2019 ESC Guidelines for the diagnosis and management of chronic coronary syndromes	Europe	/	ESC	ESC approach
Tykarski 2019	2019	2019 guidelines for the management of hypertension	Europe	Poland	PSH	Traffic light signalling system-based classification
Tran 2019	2019	New guidelines from the Thrombosis and Haemostasis Society of Australia and New Zealand for the diagnosis and management of venous thromboembolism	Oceania	Australia /New Zealand	THANZ	GRADE approach
Feitosa-Filho 2019	2019	Updated geriatric cardiology guidelines of the Brazilian society of cardiology – 2019	South America	Brazil	BSC	ESC approach
Chantrarat 2019	2019	Heart failure council of Thailand (HFCT) 2019 heart failure guideline: Atrial fibrillation in heart failure guidelines	Asia	Thailand	HFCT	ACCF/AHA approach
Arnett 2019	2019	2019 ACC/AHA Guideline on the Primary Prevention of Cardiovascular Disease: A Report of the American College of Cardiology/American Heart Association Task Force on Clinical Practice Guidelines	North America	USA	ACC/AHA	ACCF/AHA approach

CG71	2019	Familial hypercholesterolaemia: identification and management	Europe	UK	NICE	modified GRADE approach
Rosenzweig 2019	2019	Primary Prevention of ASCVD and T2DM in Patients at Metabolic Risk: An Endocrine Society Clinical Practice Guideline	North America	USA	ADA/ESE	modified GRADE approach
Nakamura 2022	2018	JCS/JSCVS 2018 Guideline on Revascularization of Stable Coronary Artery Disease	Asia	Japan	JCS/JSCVS	ACCF/AHA approach
Sousa-Uva 2019	2018	2018 ESC/EACTS Guidelines on myocardial revascularization	Europe	/	ESC/EACTS	ESC approach
Kimura 2019	2018	JCS 2018 Guideline on Diagnosis and Treatment of Acute Coronary Syndrome	Asia	Japan	JCS	ACCF/AHA approach
Li 2018	2018	2018 Guidelines of the Taiwan Society of Cardiology, Taiwan Society of Emergency Medicine and Taiwan Society of Cardiovascular Interventions for the management of non ST-segment elevation acute coronary syndrome	Asia	China	TSC/TSEM/TSCI	ESC approach
Joung 2018	2018	2018 Korean guideline of atrial fibrillation management	Asia	Korea	KHRS	ESC approach
Brieger 2018	2018	National Heart Foundation of Australia and the Cardiac Society of Australia and New Zealand: Australian Clinical Guidelines for the Diagnosis and Management of Atrial Fibrillation 2018	Oceania	Australia /New Zealand	NHFA/CSANZ	GRADE approach

AACE, American Association of Clinical Endocrinologists; ACC, American College of Cardiology; ACCF, American College of Cardiology Foundation; ACCP, American College of Clinical Pharmacy; ADA, American Diabetes Association; AHA, American Heart Association; APTH, American Physical Therapy Association; ASCO, Amercian Socirty of Clinical Oncology; ASPC, American Society for Preventive Cardiology; BCR, Brazilian College of Radiology; BSAVS, Brazilian Society of Angiology and Vascular Surgery; BSC, Brazilian Society of Cardiology; BSCO, Brazilian Society of Clinical Oncology; BSNM, Brazilian Society of Nuclear Medicine; CACM, China Association of Chinese Medicine; CAIM, Chinese Association of Integrative Medicine; C-CHANGE, Canadian Cardiovascular Harmonized National Guidelines Endeavour; CMDA, Chinese Medical Doctor Association; CSANZ, Cardiac Society of Australia and New Zealand; DGN, Deutsche Gesellschaft für Neurointensivmedizin; EACTS, European Association for Cardio-Thoracic Surgery; EAS, European Atherosclerosis Society; EHA, European Hematology Association; EHRA, European Heart Rhythm Association; ESC, European Society of Cardiology; ESE, European Society of Endocrinology; ESMO, European Society for Medical Oncology; ESTRO, European Society for Therapeutic Radiology and Oncology; ESVS, European Society for Vascular Surgery; GPAC, Guidelines and Protocols and Advisory Committee; GRADE, Grading of Recommendations Assessment, Development and Evaluation; HFCT, Heart failure council of Thailand; HFSA, Heart Failure Society of America; ICOS, International Cardio-Oncology Society; ITAC, International Initiative on Thrombosis and Cancer; JCS, Japanese Circulation Society; JHFS, Japanese Heart Failure Society; JHRS, Japanese Heart Rhythm Society; JSCVS, Japanese Society for Cardiovascular Surgery; KHRS, Korea Heart Rhythm Society; NCS, Neurocritical Care Society; NHFA, National Heart Foundation of Australia; NICE, National Institute for Clinical Excellence; NLA, National Lipid Association; PCNA, Preventive Cardiovascular Nurses Association; PSH,

Polish Society of Hypertension; SCAI, Society for Cardiovascular Angiography and Interventions; STS, Society of Thoracic Surgeons; THANZ, Thrombosis and Haemostasis Society of Australia and New Zealand; TSC, Taiwan Society of Cardiology; TSCI, Taiwan Society of Cardiovascular Interventions; TSEM, Taiwan Society of Emergency Medicine.

Supplementary Table 6 Criteria for Forming Recommendations in Included CPGs

Criteria	Class/Strength of recommendations	Quality/Level of evidence
GRADE approach	**Strong:** We recommend that the desirable effects of adherence to a recommendation outweigh the undesirable effects.<br>**Weak:** We suggest that the desirable effects of adherence to a recommendation probably outweigh the undesirable effects, but is not confident.	**High:** We are very confident that the true effect lies close to that of the estimate of the effect.<br>**Moderate:** We are moderately confident in the effect estimate: The true effect is likely to be close to the estimate of the effect, but there is a possibility that it is substantially different.<br>**Low:** Our confidence in the effect estimate is limited-The true effect maybe substantially different from the estimate of the effect.<br>**Very low:** We have very little confidence in the effect estimate-The true effect is likely to be substantially different from the estimate of effect.
ESC approach	**Class I-Is recommended or is indicated:** Evidence and/or general agreement that a given treatment or procedure is beneficial, useful, effective.<br>**Class II:** Conflicting evidence and/or a divergence of opinion about the usefulness/efficacy of the given treatment or procedure.<br>**Class IIa-Should be considered:** Weight of evidence/opinion is in favour of usefulness/efficacy.<br>**Class IIb-May be considered:** Usefulness/efficacy is less well established by evidence/opinion.<br>**Class III-Is not recommended:** Evidence or general agreement that the given treatment or procedure is not useful/effective, and in some cases may be harmful.	**Level A:** Data derived from multiple randomized clinical trials or meta-analyses.<br>**Level B:** Data derived from a single randomized clinical trial or large non-randomized studies.<br>**Level C:** Consensus of opinion of the experts and/or small studies, retrospective studies, registries.
ACCF/AHA approach	**Class 1-Strong:** Conditions for which there is evidence and/or general agreement that a given procedure or treatment is useful and effective.<br>**Class 2:** Conditions for which there is conflicting evidence and/or a divergence of opinion about the usefulness/efficacy of a procedure or treatment.<br>**2a-Moderate:** Weight of evidence/opinion is in favor of usefulness/efficacy.<br>**2b-Weak:** Usefulness/efficacy is less well established by evidence/opinion.<br>**Class 3:** Conditions for which there is evidence and/or general agreement that the procedure/treatment is not useful/effective and in some cases may be harmful.<br>**No Benefit-Moderate:** Procedure/Test not helpful or Treatment w/o established proven benefit.<br>**Harm-Strong:** Procedure/Test leads to excess cost w/o benefit or is harmful, and or Treatment is harmful.	**Level A:** 1) High-quality evidence from more than 1 RCT; 2) Meta-analyses of high-quality RCTs; 3) One more than RCTs corroborated by high-quality registry studies.<br>**Level B-R:** 1) Moderate-quality evidence from more than 1 RCT; 2) Meta-analyses of moderate-quality RCTs.<br>**Level B-NR:** 1) Moderate-quality evidence from more than 1 or more well-designed, well-executed nonrandomized studies, observational studies, or registry studies; 2) Meta-analyses of such studies.<br>**Level C-LD:** 1) Randomized or nonrandomized observational or registry studies with limitations of design or execution; 2) Meta-analyses of such studies; 3) Physiological or mechanistic studies in human subjects.<br>**Level C-EO:** Consensus of expert opinion based

		on clinical experience.
APTH approach	**A-Strong:** A preponderance of level I studies but at least 1 level I study directly on the topic supports the recommendation.<br>**B-Moderate:** A preponderance of level II studies but at least 1 level II study directly on the topic supports the recommendation.<br>**C-Weak:** A single level II study at 25% critical appraisal score or a preponderance of level III and IV studies, including statements of consensus by content experts supports the recommendation.<br>**D-Theoretical/foundational:** A preponderance of evidence from animal or cadaver studies, from conceptual/theoretical models/principles, or from basic science/bench research, or published expert opinion in peer reviewed journals supports the recommendation.<br>**P-Best practice:** Recommended practice based on current clinical practice norms, exceptional situations where validating studies have not or cannot be performed and there is a clear benefit, harm, or cost, and/or the clinical experience of the guideline development group.<br>**R-Research:** There is an absence of research on the topic, or higher-quality studies conducted on the topic disagree with respect to their conclusions. The recommendation is based on these conflicting conclusions or absent studies.	**I:** Evidence obtained from high-quality diagnostic studies, prognostic or prospective studies, cohort studies or randomized controlled trials, meta-analyses or systematic reviews (critical appraisal score 50% of criteria.<br>**II:** Evidence obtained from lesser-quality diagnostic studies, prognostic or prospective studies, cohort studies or randomized controlled trials, meta-analyses or systematic reviews (eg, weaker diagnostic criteria and reference standards, improper randomization, no blinding, 80% follow-up), (Critical appraisal score 50% of criteria).<br>**III:** Case-controlled studies or retrospective studies.<br>**IV:** Case studies and case series.<br>**V:** Expert opinion.
ESMO approach	**A:** Strong evidence for efficacy with a substantial clinical benefit, strongly recommended.<br>**B:** Strong or moderate evidence for efficacy but with a limited clinical benefit, generally recommended.<br>**C:** Insufficient evidence for efficacy or benefit does not outweigh the risk or the disadvantages (adverse events, costs, etc.), optional.<br>**D:** Moderate evidence against efficacy or for adverse outcome, generally not recommended.<br>**E:** Strong evidence against efficacy or for adverse outcome, never recommended.	**I:** Evidence from at least one large randomised, controlled trial of good methodological quality (low potential for bias) or meta-analyses of well-conducted randomised trials without heterogeneity.<br>**II:** Small randomised trials or large randomised trials with a suspicion of bias (lower methodological quality) or meta-analyses of such trials or of trials demonstrated heterogeneity.<br>**III:** Prospective cohort studies.<br>**IV:** Retrospective cohort studies or case-control studies.<br>**V:** Studies without control group, case reports, expert opinions.
Traffic light signalling system-based classification	**Green:** a given management approach is recommended, generally based on clear evidence from research studies, or unequivocal expert opinion reflecting current clinical practice.<br>**Yellow:** a given management approach is suggested as appropriate despite weaker supporting data due to lacking or equivocal evidence from research studies, based on the opinion of the majority of experts reflecting common sense and their personal clinical experience.	/

	**Red:** a given management approach should be abandoned as it is considered not justified or harmful, generally based on clear evidence from research studies or the opinion of the majority of experts.	

ACCF, American College of Cardiology Foundation; AHA, American Heart Association; APTH, American Physical Therapy Association; ESC, European Society of Cardiology; ESMO, European Society for Medical Oncology; GRADE, Grading of Recommendations Assessment, Development and Evaluation.

Supplementary Table 7 Overview of Recommendations on Prediction Models

Guideline ID	Overview of recommendations	Relevant model	Class/Strength of recommendations	Quality/Level of evidence
Virani 2023	R1 (support): In patients with CCD, it is recommended that risk stratification incorporate all available information, including noninvasive, invasive, or both cardiovascular diagnostic testing results or use validated risk scores to classify patients as low (<1%), intermediate (1%-3%), or high (>3%) yearly risk for cardiovascular death or nonfatal MI.<br>R2 (support): Women with CCD who are contemplating pregnancy or who are pregnant should be risk-stratified and counseled regarding risks of adverse maternal, obstetric, and fetal outcomes.	PROMISE Minimal-Risk Tool (R1)<br>Post–treadmill test mortality prediction rule (R1)<br>CARPREG II risk score (R2)	R1: 1<br>R2: 1	R1: B-NR<br>R2: C-LD
Ueng 2023	R1 (support): For adults 40 to 75 years of age without established ASCVD, chronic inflammatory diseases, diabetes, CKD (eGFR < 60 mL/min/1.73 m2), or a family history of premature MI, clinicians should consider assessing traditional risk factors and calculate the 10-year risk of CAD by using the TwCCCC risk charts.	TwCCCC risk charts (R1)	R1: IIa	R1: B
Takase 2023	R1 (support): Clinical diagnosis of congenital LQTS is made with an LQTS risk score ≥3.5 after excluding secondary LQTS (Schwartz score).	Schwartz score (R1)	R1: I	R1: C
Rajajee 2023	R1 (nonsupport): When counseling family members or surrogates of comatose survivors of IHCA, we suggest the GOFAR clinical prediction model alone not be considered a reliable predictor of poor functional outcome assessed at 3 months or later.	GO-FAR score (R1)	R1: Weak	R1: Moderate
Key 2023	R1 (support): There is substantial variation in risk of VTE between individual patients with cancer and cancer settings. Patients with cancer should be assessed for VTE risk initially and periodically thereafter, particularly when starting systemic antineoplastic therapy or at the time of hospitalization. Individual risk factors, including biomarkers or cancer site, do not reliably identify patients with cancer at high risk of VTE. In the ambulatory setting among patients with solid tumors treated with systemic therapy, risk assessment can be conducted on the basis of a validated risk assessment tool (Khorana score).	KRS (R1)	R1: Strong	R1: Moderate
Hiraoka 2023	R1 (support): Use of the RCRI is recommended for preoperative risk assessment.	RCRI (R1)	R1: I	R1: B
Falanga 2023	R1 (support): VTE risk assessment should be based on validated RAMs such as the KRS, the Prospective	KRS (R1)	R1: C	R1: III

	Comparison of Methods for thromboembolic risk assessment with clinical Perceptions and AwareneSS in real-life patients-CancerAssociated Thrombosis (COMPASS-CAT) or the Vienna Cancer and Thrombosis Study (Vienna-CATS) nomogram score.<br>R2 (support): An estimated risk of VTE >8%-10% at 6 months is suggested as threshold for discussing primary thromboprophylaxis . This risk is observed in patients with a KRS ≥ 2 and can individually be calculated with the Vienna-CATS nomogram score and the COMPASS-CAT score.	COMPASS-CAT RAM(R1; R2)<br>Vienna-CATS score (R1; R2)	R2: C	R2: II
Byrne 2023	R1 (support): The use of established risk scores (e.g. GRACE risk score) for prognosis estimation should be considered.	GRACE risk score 2.0 (R1)	R1: IIa	R1: B
Arbelo 2023	R1 (support): The HCM Risk-SCD calculator is recommended as a method of estimating risk of sudden death at 5 years in patients aged ≥16 years for primary prevention.<br>R2 (support): Validated paediatric-specific risk prediction models (e.g. HCM Risk-Kids) are recommended as a method of estimating risk of sudden death at 5 years in patients aged <16 years for primary prevention.<br>R3 (support): In patients with LV apical aneurysms, decisions about primary prevention ICD based on an assessment of risk using the HCM Risk-SCD or a validated paediatric risk-prediction (e.g. HCM Risk-Kids) tool and not solely on the presence of the aneurysm should be considered.<br>R4 (support): The updated 2019 ARVC risk calculator should be considered to aid individualized decision-making for ICD implantation in patients with ARVC.	HCM Risk-SCD calculator (R1; R3)<br>HCM Risk-Kids calculator (R2; R3)<br>ARVC risk calculator 2.0 (R4)	R1: I<br>R2: I<br>R3: IIa<br>R4: IIa	R1: B<br>R2: B<br>R3: B<br>R4: B
Zeppenfeld 2022	R1 (support): In LQTS, it should be considered to calculate the arrhythmic risk before initiation of therapy based on the genotype and the duration of QTc interval.<br>R2 (support): ICD implantation may be considered in asymptomatic LQTS patients with high-risk profile (according to the 1-2-3 LQTS Risk calculator) in addition to genotype-specific medical therapies (mexiletine in LQT3 patients).	1-2-3 LQTS Risk calculator (R1; R2)	R1: IIa<br>R2: IIb	R1: C<br>R2: B
Nakamura 2022	R1 (support): JapanSCORE and STS score are recommended for prediction of in-hospital mortality rate of coronary artery bypass grafting.<br>R2 (support): SYNTAX score II is recommended for treatment decision-making in multivessel disease.<br>R3 (support): J-CTO score is recommended for assessment of the difficulty of PCI in CTO lesion using.	JapanSCORE (R1)<br>STS risk score (R1)<br>SYNTAX score II (R2)<br>J-CTO score (R3)	R1: IIa<br>R2: IIa<br>R3: IIa	R1: C<br>R2: B<br>R3: B

Lyon 2022	R1 (support): Baseline CV risk assessmentc and estimation of 10-year fatal and non-fatal CVD risk with SCORE2 or SCORE2-OP is recommended in patients treated with ADT without pre-existing CVD.<br>R2 (support): Baseline CV risk assessmentc and estimation of 10-year fatal and non-fatal CVD risk with SCORE2 or SCORE2-OP is recommended in BC patients receiving endocrine therapies without pre-existing CVD.<br>R3 (support): Annual CV risk assessmentc is recommended during endocrine therapy in BC patients with high 10-year risk of (fatal and non-fatal) CV events according to SCORE2/SCORE2-OP.<br>R4 (support): CV risk assessmentc should be considered every 5 years in BC patients with low or moderate 10-year risk of (fatal and non-fatal) CV events according to SCORE2/SCORE2-OP.<br>R5 (support): Baseline CV risk assessmentc and estimation of 10-year fatal and non-fatal CVD risk with SCORE2 or SCORE2-OP is recommended.<br>R6 (support): CHA_2DS_2-VASc score should be considered for risk stratification for stroke/systemic thromboembolism taking into account that it may underestimate the actual thromboembolic risk.	SCORE2 (R1; R2; R3; R4; R5)<br>SCORE2-OP (R1; R2; R3; R4; R5)<br>CHA_2DS_2-VASc score (R6)	R1: I<br>R2: I<br>R3: I<br>R4: IIa<br>R5: I<br>R6: IIa	R1: B<br>R2: C<br>R3: C<br>R4: C<br>R5: B<br>R6: C
Jain 2022	R1 (support): We recommend that a CV risk assessment be completed every 5 years for men and women aged 40 to 75 yr using the modified FRS or CLEM to guide therapy to reduce major CV events. A risk assessment might also be completed whenever a patient’s expected risk status changes.	FRS (R1)	R1: Strong	R1: High
Hillegass 2022	R1 (support): During initial interview and physical examination assess risk of VTE in patients with reduced mobility (Padua Prediction Score or Caprini score).<br>R2 (support): When a patient presents with conditions (ie, cancer or inherited clotting disorder) that independently increase VTE risk, therapists should have a high index of suspicion for VTE and assess for additional risk factors (KRS).	Padua Prediction Score (R1)<br>Caprini score (R1)<br>KRS (R2)	R1: A-Strong<br>R1: B-Moderate	R1: I<br>R2: I
Heidenreich 2022	R1 (support): In ambulatory or hospitalized patients with HF, validated multivariable risk scores can be useful to estimate subsequent risk of mortality (Seattle Heart Failure Model; Heart Failure Survival Score; MAGGIC; CHARM Risk Score; CORONA Risk Score; PARADIGM-HF; HF-ACTION; GUIDE-IT; I-PRESERVE Score; TOPCAT; ADHERE Classification and Regression Tree (CART) Model; GWTG-HF Risk Score; EFFECT Risk Score; ESCAPE Risk Model and Discharge Score).<br>R2 (support): In the general population, validated multivariable risk scores can be useful to estimate subsequent risk	Seattle Heart Failure Model (R1)<br>Heart Failure Survival Score (R1)<br>MAGGIC (R1)<br>CHARM Risk Score (R1)<br>CORONA Risk Score (R1)<br>PARADIGM-HF (R1)	R1: 2a<br>R2: 2a	R1: B-NR<br>R2: B-NR

	of incident HF (Framingham Heart Failure Risk Score; Health ABC Heart Failure Score; ARIC Risk Score; PCP-HF).	HF-ACTION (R1)<br>GUIDE-IT (R1)<br>I-PRESERVE Score (R1)<br>TOPCAT (R1)<br>ADHERE Classification and Regression Tree (CART) Model (R1)<br>GWTG-HF Risk Score (R1)<br>EFFECT Risk Score (R1)<br>ESCAPE Risk Model and Discharge Score (R1)<br>Framingham Heart Failure Risk Score (R2)<br>Health ABC Heart Failure Score (R2)<br>ARIC Risk Score (R2)<br>PCP-HF (R2)		
Farge 2022	R1 (support): Primary prophylaxis with direct oral anticoagulant(rivaroxaban or apixaban) is recommended in ambulatory patients who are receiving systemic anticancer therapy and are at intermediateto-high-risk of VTE, identified by a validated risk assessment model (ie, a Khorana score ≥2), and not actively bleeding or not at a high risk for bleeding.	KRS (R1)	R1: Strong	R1: Moderate
Albricker 2022	R1 (support): The indication of imaging methods for the diagnosis of acute PE should be based on hemodynamic status and clinical assessment of pretest probability, with the application of validated rules (Wells score, Geneva score, PERC criteria) combined with D-dimer. The aim is to avoid unnecessary use of imaging methods.	Wells score (R1)<br>Geneva score (R1)<br>PERC rule (R1)	R1: Strong	R1: High
Visseren 2021	R1 (support): In apparently healthy people <70 years without established ASCVD, DM, CKD, genetic/rarer lipid or BP disorders, estimation of 10-year fatal and non-fatal CVD risk with SCORE2 is recommended.	SCORE2 (R1)<br>SCORE2-OP (R2)	R1: I<br>R2: I	R1: B<br>R2: B

	R2 (support): In apparently healthy people >_70 years without established ASCVD, DM, CKD, genetic/rarer lipid or BP disorders, estimation of 10-year fatal and non-fatal CVD risk with SCORE2-OP is recommended.			
Tsutsui 2021	R1 (support): Evaluation concerning anticoagulant therapy by the CHADS2 score and HAS-BLED score is recommended for atrial fibrillation complicating heart failure.	CHADS2 score (R1)<br>HAS-BLED score (R1)	R1: I	R1: B
Otto 2021	R1 (support): For patients with VHD for whom intervention is contemplated, individual risks should be calculated for specific surgical and/or transcatheter procedures, using online tools when available, and discussed before the procedure as a part of a shared decision-making process (STS risk score; EuroSCORE II risk calculator; TVT Registry model; Emory risk score; Risk prediction model for in-hospital stroke after TAVR).	STS risk score (R1)<br>EuroSCORE II (R1)<br>TVT Registry model (R1)<br>Emory risk score (R1)<br>Risk prediction model for in-hospital stroke after TAVR (R1)	R1: 1	R1: CE-O
Lawton 2021	R1 (support): In patients who are being considered for CABG, calculation of the STS risk score is recommended to help stratify patient risk.<br>R2 (support): In patients with multivessel CAD, an assessment of CAD complexity, such as the SYNTAX score, may be useful to guide revascularization.	STS risk score (R1)<br>SYNTAX score II (R2)	R1: 1<br>R2: 2b	R1: B-NR<br>R2: B-NR
Kakkos 2021	R1 (nonsupport): In pregnant women with suspected deep vein thrombosis, the use of D dimer and Wells score is not recommended.	Wells score (R1)	R1: III	R1: B
Hindricks 2021	R1 (support): For stroke risk assessment, a risk-factor-based approach is recommended, using the CHA_2DS_2-VASc clinical stroke risk score to initially identify patients at 'low stroke risk' (CHA_2DS_2-VASc score = 0 in men, or 1 in women) who should not be offered antithrombotic therapy.<br>R2 (support): For a formal risk-score-based assessment of bleeding risk, the HAS-BLED score should be considered to help address modifiable bleeding risk factors, and to identify patients at high risk of bleeding (HAS-BLED score >_3) for early and more frequent clinical review and follow-up.<br>R3 (support): In patients with AHRE/subclinical AF detected by CIED or insertable cardiac monitor, it is recommended to conduct complete cardiovascular evaluation with ECG recording, clinical risk factors/comorbidity evaluation, and thrombo-embolic risk assessment using the CHA_2DS_2-VASc score.	CHA_2DS_2-VASc score (R1; R3)<br>HAS-BLED score (R2)	R1: I<br>R2: IIa<br>R3: I	R1: A<br>R2: B<br>R3: B
Mach 2020	R1 (support): Total risk estimation using a risk estimation system such as SCORE is recommended for	SCORE (R1)	R1: I	R1: C

	asymptomatic adults >40 years of age without evidence of CVD, DM, CKD, familial hypercholesterolaemia, or LDL-C >4.9 mmol/L (>190 mg/dL).<br>R2 (nonsupport): Risk scores developed for the general population are not recommended for CV risk assessment in patients with DM or FH.	SCORE (R2)	R2: III	R2: C
Liao 2020	R1 (support): Preliminary risk stratification should be conducted according to AMI patient's ST-segement in ECG. For NSTEMI patients, GRACE risk score and TIMI risk score are commonly used to further evaluate the risk of ischemia.	GRACE risk score 2.0 (R1)<br>TIMI risk score (R1)	R1: Strong	R1: High
Knuuti 2020	R1 (support): Total risk estimation using a risk-estimation system such as SCORE is recommended for asymptomatic adults >40 years of age without evidence of CVD, diabetes, CKD, or familial hypercholesterolaemia.	SCORE (R1)	R1: I	R1: C
Hajjar 2020	R1 (support): Cancer patients should have their risk for VTE assessed on an outpatient basis with the Khorana or the CAT score, and the benefits and risks of that strategy should be analyzed on a case-by-case basis, because they are associated with a reduction in thromboembolic events but not in mortality.	KRS (R1)<br>Vienna-CATS score (R1)	R1: IIa	R1: B
Tykarski 2019	R1 (support): Assessment of the global cardiovascular risk using the Framingham risk score and the SCORE risk estimation chart (recalibrated nationally as the Pol-SCORE risk chart) is recommended in patients with hypertension to determine indications for non-blood pressure lowering treatment.	FRS (R1)<br>SCORE (R1)	Green	/
Tran 2019	R1 (support): A non-high pre-test probability (Wells or Geneva score) combined with a negative D-dimer result safely excludes VTE without imaging.<br>R2 (support): PE can be excluded without D-dimer or radiological testing in selected patients if the PE rule-out criteria (negative PERC rule) are met.	Wells score (R1)<br>Geneva score (R1)<br>PERC rule (R2)	R1: Strong<br>R2: Strong	R1: High<br>R2: Moderate
Sousa-Uva 2019	R1 (support): It is recommended that the STS score is calculated to assess in-hospital or 30-day mortality, and in-hospital morbidity after CABG.<br>R2 (support): Calculation of the EuroSCORE II score may be considered to assess in-hospital mortality after CABG.	STS risk score (R1)<br>EuroSCORE II (R2)	R1: I<br>R2: IIb	R1: B<br>R2: B
Kimura 2019	R1 (support): Risk assessment using risk scores (GRACE, TIMI, etc.) should be performed at the Time of Initial Diagnosis.<br>R2 (support): Using a risk score (GRACE, TIMI, etc.) to determine treatment strategy should be considered at the	GRACE risk score 2.0 (R1; R2; R3)<br>TIMI risk score (R1; R2; R3)	R1: I<br>R2: IIa<br>R3: IIa	R1: A<br>R2: B<br>R3: B

	Time of Initial Diagnosis.<br>R3 (support): Use of risk scores such as TIMI and GRACE should be considered in NSTE-ACS.			
Feitosa-Filho 2019	R1 (support): The HAS-BLED score is recommended to evaluate risk of bleeding during anticoagulation.	HAS-BLED score (R1)	R1: I	R1: B
Chantrarat 2019	R1 (support): Risk stratification by CHA_2DS_2-VASc and HAS-BLED scores is recommended for stroke prevention in patients with AF in HF being treated with oral anticoagulant (OAC) and left atrial appendage (LAA) occluder device.	CHA_2DS_2-VASc score (R1)<br>HAS-BLED score (R1)	R1: I	R1: A
Arnett 2019	R1 (support): For adults 40 to 75 years of age, clinicians should routinely assess traditional cardiovascular risk factors and calculate 10-year risk of ASCVD by using the pooled cohort equations (PCE).	PCE (R1)	R1: 1	R1: B-NR
Li 2018	R1 (support): The established scoring systems, such as TIMI or GRACE risk scores can be used for risk assessment in NSTE-ACS.	GRACE risk score 2.0 (R1)<br>TIMI risk score (R1)	R1: I	R1: B
Joung 2018	R1 (support): The CHA_2DS_2-VASc score is recommended for stroke risk prediction in patients with AF.<br>R2 (support): The HAS-BLED score is recommended to address modifiable bleeding risk factors in all AF patients. Those potentially at high risk (HAS-BLED score ≥3) warrant more frequent and regular reviews or follow-up.	CHA_2DS_2-VASc score (R1)<br>HAS-BLED score (R2)	R1: I<br>R2: I	R1: A<br>R2: A
Brieger 2018	R1 (support): The CHA2DS2-VA score—the sexless CHA_2DS_2-VASc score—is recommended for predicting stroke risk in AF.<br>R2 (support): The CHA2DS2-VA score should be re-evaluated yearly in low-risk patients who are not anticoagulated.	CHA_2DS_2-VASc score (R1; R2)	R1: Strong<br>R2: Strong	R1: Moderate<br>R2: Low
NG238	R1 (support): Use the QRISK3 tool to calculate the estimated CVD risk within the next 10 years for people aged between 25 and 84 without CVD.<br>R2 (support): Use the QRISK3 tool for people with type 2 diabetes aged between 25 and 84.<br>R3 (nonsupport): Do not use a risk assessment tool for people who are at high risk of CVD, including people with: 1) type 1 diabetes; 2) an estimated glomerular filtration rate less than 60 ml/min/1.73 m^2 and/or albuminuria; 3) familial hypercholesterolaemia or other inherited disorders of lipid metabolism.<br>R4 (support): Consider using a lifetime risk tool such as QRISK3-lifetime to inform discussions on CVD risk and to motivate lifestyle changes, particularly for people with a 10-year QRISK3 score less than 10%, and people under 40	QRISK3 tool (R1; R2; R4)<br>QRISK3 tool (R3)	R1: Strong<br>R2: Strong<br>R3: Strong<br>R4: Strong	/

	who have CVD risk factors.			
CG71	R1 (nonsupport): Coronary heart disease risk estimation tools, such as QRISK2 and those based on the Framingham algorithm, should not be used because people with FH are already at a high risk of premature coronary heart disease.	QRISK2 (R1)<br>FRS (R1)	R1: Strong	/
NG156	R1 (nonsupport): Do not use the following risk assessment tools to determine whether or not repair is suitable for a person with an asymptomatic unruptured AAA: 1) British Aneurysm Repair score; 2) Carlisle Calculator; 3) Comorbidity Severity Score; 4) Glasgow Aneurysm Scale; 5) Medicare risk prediction tool; 6) Modified Leiden score; 7) Physiological and Operative Severity Score for enUmeration of Mortality (POSSUM); 8) Vascular-POSSUM; 9) Vascular Biochemical and Haematological Outcome Model (VBHOM); 10) Vascular Governance North West (VGNW) risk model.	British Aneurysm Repair score (R1)<br>Carlisle Calculator (R1)<br>Comorbidity Severity Score (R1)<br>Glasgow Aneurysm Scale (R1)<br>Medicare risk prediction tool (R1)<br>Modified Leiden score (R1)<br>Physiological and Operative Severity Score for enUmeration of Mortality (POSSUM) (R1)<br>Vascular-POSSUM (R1)<br>Vascular Biochemical and Haematological Outcome Model (VBHOM) (R1)<br>Vascular Governance North West (VGNW) risk model (R1)	R1: Strong	/
NG158	R1 (support): If DVT is suspected, use the 2-level DVT Wells score (table 1) to estimate the clinical probability of DVT.<br>R2 (support): If PE is suspected, use the 2-level PE Wells score (table 2) to estimate the clinical probability of PE.<br>R3 (support): If clinical suspicion of PE is low based on the overall clinical impression (from general medical history, physical examination and any initial investigations such as electrocardiography or chest X-ray), and other diagnoses are feasible, consider using the pulmonary embolism rule-out criteria (PERC) to help determine whether any further investigations for PE are needed.	Wells score (R1; R2)<br>PERC rule (R3)	R1: Strong<br>R2: Strong<br>R3: Strong	/

NG185	R1 (support): As soon as the diagnosis of unstable angina or NSTEMI is made, and aspirin and antithrombin therapy have been offered, formally assess individual risk of future adverse cardiovascular events using an established risk scoring system that predicts 6-month mortality (for example, Global Registry of Acute Cardiac Events [GRACE]).	GRACE risk score 2.0 (R1)	R1: Strong	/
NG196	R1 (support): Use the CHA_2DS_2-VASc stroke risk score to assess stroke risk in people with any of the following: 1) symptomatic or asymptomatic paroxysmal, persistent or permanent atrial fibrillation 2) atrial flutter; 3) a continuing risk of arrhythmia recurrence after cardioversion back to sinus rhythm or catheter ablation.<br>R2 (support): Use the ORBIT bleeding risk score for assessing the risk of bleeding when: 1) considering starting anticoagulation in people with atrial fibrillation; and 2) reviewing people already taking anticoagulation.	CHA_2DS_2-VASc score (R1)<br>ORBIT bleeding risk score (R2)	R1: Strong<br>R2: Strong	/
Newman 2020	R1 (support): In adults with endocrine disorders, we recommend conducting a cardiovascular risk assessment by evaluating traditional risk factors, including the calculation of 10-year atherosclerotic cardiovascular disease risk using a tool such as the Pooled Cohort Equations.<br>R2 (support): In individuals who have obesity, we recommend the assessment of 10-year risk for atherosclerotic cardiovascular disease to guide the use of lipidlowering therapy (PCE).	PCE (R1; R2)	R1: Strong<br>R2: Strong	R1: Moderate<br>R2: Moderate
Rosenzweig 2019	R1 (support): In individuals identified as having metabolic risk, we recommend global assessment of 10-year risk for either coronary heart disease or atherosclerotic cardiovascular disease to guide the use of medical or pharmacological therapy (PCE; FRS; SCORE; PROCAM score).<br>R2 (support): In individuals 40 to 75 years of age with lowdensity lipoprotein cholesterol 70 to 189 mg/dL (1.8 to 4.9 mmol/L), we recommend a 10-year risk for atherosclerotic cardiovascular disease should be calculated (PCE; FRS; SCORE; PROCAM score).	PCE (R1; R2)<br>FRS (R1; R2)<br>SCORE (R1; R2)<br>PROCAM score (R1; R2)	R1: Strong<br>R2: Strong	R1: Moderate<br>R2: Moderate
BCG 2021	R1 (support): Consider to assess CVD risk in: 1) all asymptomatic men and women ≥40 years; 2) all patients with pre-existing risk-related conditions (e.g., Hypertension, DM, CKD); and 3) all patients with a known family history of premature CVD (defined as men aged <55 years and women aged <65 years in first degree relatives) (FRS).	FRS (R1)	R1: Strong	R1: High

ADHERE, Acute Decompensated Heart Failure National Registry; ARIC, Atherosclerosis Risk in Communities; CARPREG, Cardiac Disease in Pregnancy Study; CART, Classification and Regression Tree; CHADS2, Congestive Heart Failure, Hypertension, Age > 75 Years, Diabetes, Stroke/Transient Ischemic Attack; CHA_2DS_2-VASc, Congestive heart failure, Hypertension, Age ≥ 75 years (2 points), Diabetes mellitus, Stroke (2 points)-Vascular disease, Age 65-74 years, Sex category (female); CHARM,

Candesartan in Heart failure-Assessment of Reduction in Mortality and morbidity; COMPASS-CAT, Prospective Comparison of Methods for thromboembolic risk assessment with clinical Perceptions and AwareneSS in real-life patients-Cancer Associated Thrombosis; CORONA, Controlled Rosuvastatin Multinational Trial in Heart Failure; EFFECT, Enhanced Feedback for Effective Cardiac Treatment; ESCAPE, Evaluation Study of Congestive Heart Failure and Pulmonary Artery Catheterization Effectiveness; FRS, Framingham Risk Score; GO-FAR, Good Outcome Following Attempted Resuscitation; GRACE, Global Registry of Acute Coronary Events; GUIDE-IT, Guiding Evidence-Based Therapy Using Biomarker Intensified Treatment; GWTG-HF, Get With The Guidelines–Heart Failure; HAS-BLED, Hypertension, Abnormal renal/liver function, Stroke, Bleeding history or predisposition, Labile international normalized ratio, Elderly (> 65 years), Drugs/alcohol concomitantly; HF, heart failure; HF-ACTION, Heart Failure: A Controlled Trial Investigating Outcomes of Exercise Training; HFSA, Heart Failure Society of America; I-PRESERVE, Irbesartan in Heart Failure with Preserved Ejection Fraction Study; J-CTO, Multicenter Chronic Total Occlusion Registry in Japan; KRS, Khorana risk score; LQTS, long QT syndrome; MAGGIC, Meta-analysis Global Group in Chronic Heart Failure; PARADIGM-HF, Prospective Comparison of ARNI With ACEI to Determine Impact on Global Mortality and Morbidity in Heart Failure trial; PCE, Pooled Cohort Equations; PCP-HF, Pooled Cohort Equations to Prevent HF; PERC, Pulmonary Embolism Rule-Out Criteria; POSSUM, Physiological and Operative Severity Score for enUmeration of Mortality; PROCAM, Prospective Cardiovascular Münster; PROMISE, Prospective Multicenter Imaging Study for Evaluation of Chest Pain; RAM, risk assessment model; RCRI, Revised Cardiac Risk Index; SCORE, Systematic Coronary Risk Estimation; SCORE2, Systematic Coronary Risk Estimation 2; SCORE2-OP, Systematic Coronary Risk Estimation 2-Older Persons; STS, Society of Thoracic Surgeons; SYNTAX, Synergy between Percutaneous Coronary Intervention with TAXUS and Cardiac Surgery; TAVR, transcatheter aortic valve replacement; TIMI, Thrombolysis in Myocardial Infarction; TOPCAT, Treatment of Preserved Cardiac Function Heart Failure with an Aldosterone Antagonist trial; TVT, Transcatheter Valve Therapy; TwCCCC, Taiwan Chin-Shan Community Cardiovascular Cohort; VBHOM, Vascular Biochemical and Haematological Outcome Model; VGNW, Vascular Governance North West; Vienna-CATS, Vienna Cancer and Thrombosis Study.

Supplementary Table 8 Primary Studies of Guideline-recommended Prediction Models

**Study ID**	**Model Name**	**Article Title**	**Publication Title**	**DOI**
Cadrin-Tourigny 2022	ARVC risk calculator 2.0	A new prediction model for ventricular arrhythmias in arrhythmogenic right ventricular cardiomyopathy	European Heart Journal	10.1093/eurheartj/ehac180
Mazzanti 2022	1-2-3 LQTS Risk calculator	Independent validation and clinical implications of the risk prediction model for long QT syndrome (1-2-3-LQTS-Risk)	Europace	10.1093/europace/euab238
de Vries 2021	SCORE2-OP	SCORE2-OP risk prediction algorithms: estimating incident cardiovascular event risk in older persons in four geographical risk regions	European Heart Journal	10.1093/eurheartj/ehab312
Hageman 2021	SCORE2	SCORE2 risk prediction algorithms: new models to estimate 10-year risk of cardiovascular disease in Europe	European Heart Journal	10.1093/eurheartj/ehab309
Angraal 2020	TOPCAT	Machine learning prediction of mortality and hospitalization in heart failure with preserved ejection fraction	JACC: Heart Failure	10.1016/j.jchf.2019.06.013
Simpson 2020	PARADIGM-HF	Prognostic models derived in PARADIGM-HF and validated in ATMOSPHERE and the Swedish Heart Failure Registry to predict mortality and morbidity in chronic heart failure	JAMA Cardiology	10.1001/jamacardio.2019.5850
Cronin 2019	Caprini score	Completion of the Updated Caprini Risk Assessment Model (2013 Version)	Clinical and Applied Thrombosis-Hemostasis	10.1177/1076029619838052
Khan 2019	PCP-HF	10-year risk equations for incident heart failure in the general population	Journal of the American College of Cardiology	10.1016/j.jacc.2019.02.057
Kiani 2019	Emory risk score	Development of a Risk Score to Predict New Pacemaker Implantation After Transcatheter Aortic Valve Replacement	JACC: Cardiovascular Interventions	10.1016/j.jcin.2019.07.015
Norrish 2019	HCM Risk-Kids calculator	Development of a Novel Risk Prediction Model for Sudden Cardiac Death in Childhood Hypertrophic Cardiomyopathy (HCM Risk-Kids)	JAMA Cardiology	10.1001/jamacardio.2019.2861
O'Connor 2019	GUIDE-IT	Clinical factors related to morbidity and mortality in high-risk heart failure patients: the GUIDE-IT predictive model and risk score	European Journal of Heart Failure	10.1002/ejhf.1450
Thourani 2019	Risk prediction model for in-hospital	Development and Application of a Risk Prediction Model for In-Hospital Stroke After Transcatheter Aortic Valve Replacement: A Report From The Society of Thoracic	Annals of Thoracic Surgery	10.1016/j.athoracsur.2018.11.013

	stroke after TAVR	Surgeons/American College of Cardiology Transcatheter Valve Therapy Registry		
O'Brien 2018	STS risk score	The Society of Thoracic Surgeons 2018 Adult Cardiac Surgery Risk Models: Part 2-Statistical Methods and Results	Annals of Thoracic Surgery	10.1016/j.athoracsur.2018.03.003
Silversides 2018	CARPREG II risk score	Pregnancy Outcomes in Women With Heart Disease: The CARPREG II Study	Journal of the American College of Cardiology	10.1016/j.jacc.2018.02.076
Fordyce 2017	PROMISE Minimal-Risk Tool	Identification of Patients With Stable Chest Pain Deriving Minimal Value From Noninvasive Testing: The PROMISE Minimal-Risk Tool, A Secondary Analysis of a Randomized Clinical Trial	JAMA Cardiology	10.1001/jamacardio.2016.5501
Gerotziafas 2017	COMPASS-CAT RAM	A Predictive Score for Thrombosis Associated with Breast, Colorectal, Lung, or Ovarian Cancer: The Prospective COMPASS-Cancer-Associated Thrombosis Study	Oncologist	10.1634/theoncologist.2016-0414
Hippisley-Cox 2017	QRISK3 tool	Development and validation of QRISK3 risk prediction algorithms to estimate future risk of cardiovascular disease: prospective cohort study	British Medical Journal	10.1136/bmj.j2099
Edwards 2016	TVT Registry model	Development and Validation of a Risk Prediction Model for In-Hospital Mortality After Transcatheter Aortic Valve Replacement	JAMA Cardiology	10.1001/jamacardio.2015.0326
Fox 2014	GRACE risk score 2.0	Should patients with acute coronary disease be stratified for management according to their risk? Derivation, external validation and outcomes using the updated GRACE risk score	BMJ Open	10.1136/bmjopen-2013-004425
Goff 2014	PCE	2013 ACC/AHA guideline on the assessment of cardiovascular risk: a report of the American College of Cardiology/American Heart Association Task Force on Practice Guidelines	Circulation	10.1161/01.cir.0000437741.48606.98
O’Mahony 2014	HCM Risk-SCD calculator	A novel clinical risk prediction model for sudden cardiac death in hypertrophic cardiomyopathy (HCM risk-SCD)	European Heart Journal	10.1093/eurheartj/eht439
O’Brien 2015	ORBIT bleeding risk score	The ORBIT bleeding score: a simple bedside score to assess bleeding risk in atrial fibrillation	European Heart Journal	10.1093/eurheartj/ehv476
Farooq 2013	SYNTAX score II	Anatomical and clinical characteristics to guide decision making between coronary artery bypass surgery and percutaneous coronary intervention for individual patients: development and validation of SYNTAX score II	Lancet	10.1016/s0140-6736(13)60108-7
Pocock 2013	MAGGIC	Predicting survival in heart failure: a risk score based on 39 372 patients from 30 studies	European Heart Journal	10.1093/eurheartj/ehs337
Agarwal 2012	ARIC Risk Score	Prediction of incident heart failure in general practice: the Atherosclerosis Risk in Communities	Circulation: Heart Failure	10.1161/CIRCHEARTFAILU

		(ARIC) Study		RE.111.964841
Chien 2012	TwCCCC risk charts	Constructing a point-based prediction model for the risk of coronary artery disease in a Chinese community: a report from a cohort study in Taiwan	International Journal of Cardiology	10.1016/j.ijcard.2012.03.017
Nashef 2012	EuroSCORE II	EuroSCORE II	European Journal of Cardio-Thoracic Surgery	10.1093/ejcts/ezs043
O'Connor 2012	HF-ACTION	Factors related to morbidity and mortality in patients with chronic heart failure with systolic dysfunction: the HF-ACTION predictive risk score model	Circulation: Heart Failure	10.1161/CIRCHEARTFAILURE.111.963462
Komajda 2011	I-PRESERVE Score	Factors associated with outcome in heart failure with preserved ejection fraction: findings from the Irbesartan in Heart Failure with Preserved Ejection Fraction Study (I-PRESERVE)	Circulation: Heart Failure	10.1161/CIRCHEARTFAILURE.109.932996
Miyata 2011	JapanSCORE	Risk models including high-risk cardiovascular procedures: clinical predictors of mortality and morbidity	European Journal of Cardio-Thoracic Surgery	10.1016/j.ejcts.2010.08.050
Morino 2011	J-CTO score	Predicting successful guidewire crossing through chronic total occlusion of native coronary lesions within 30 minutes: the J-CTO (Multicenter CTO Registry in Japan) score as a difficulty grading and time assessment tool	JACC: Cardiovascular Interventions	10.1016/j.jcin.2010.09.024
Schwartz 2011	Schwartz score	QTc behavior during exercise and genetic testing for the long-QT syndrome	Circulation	10.1161/CIRCULATIONAHA.111.062182
Ay 2010	Vienna-CATS score	Prediction of venous thromboembolism in cancer patients	Blood	10.1182/blood-2010-02-270116
Barbar 2010	Padua Prediction Score	A risk assessment model for the identification of hospitalized medical patients at risk for venous thromboembolism: the Padua Prediction Score	Journal of Thrombosis and Haemostasis	10.1111/j.1538-7836.2010.04044.x
Lip 2010	CHA_2DS_2-VASc score	Refining clinical risk stratification for predicting stroke and thromboembolism in atrial fibrillation using a novel risk factor-based approach: the euro heart survey on atrial fibrillation	Chest	10.1378/chest.09-1584
O'Connor 2010	ESCAPE Risk Model and Discharge Score	Triage after hospitalization with advanced heart failure: the ESCAPE (Evaluation Study of Congestive Heart Failure and Pulmonary Artery Catheterization Effectiveness) risk model and discharge score	Journal of the American College of Cardiology	10.1016/j.jacc.2009.08.083
Peterson 2010	GWTG-HF Risk	A validated risk score for in-hospital mortality in patients with heart failure from the American	Circulation: Cardiovascular	10.1161/CIRCOUTCOMES.

	Score	Heart Association Get With The Guidelines program	Quality and Outcomes	109.854877
Pisters 2010	HAS-BLED score	A novel user-friendly score (HAS-BLED) to assess 1-year risk of major bleeding in patients with atrial fibrillation: the Euro Heart Survey	Chest	10.1378/chest.10-0134
Wedel 2009	CORONA Risk Score	Predictors of fatal and non-fatal outcomes in the Controlled Rosuvastatin Multinational Trial in Heart Failure (CORONA): incremental value of apolipoprotein A-1, high-sensitivity C-reactive peptide and N-terminal pro B-type natriuretic peptide	European Journal of Heart Failure	10.1093/eurjhf/hfn046
Butler 2008	Health ABC Heart Failure Score	Incident heart failure prediction in the elderly: the health ABC heart failure score	Circulation: Heart Failure	10.1161/CIRCHEARTFAILURE.108.768457
D'Agostino 2008	FRS	General cardiovascular risk profile for use in primary care: the Framingham Heart Study	Circulation	10.1161/circulationaha.107.699579
Khorana 2008	KRS	Development and validation of a predictive model for chemotherapy-associated thrombosis	Blood	10.1182/blood-2007-10-116327
Lauer 2007	Post–treadmill test mortality prediction rule	An externally validated model for predicting long-term survival after exercise treadmill testing in patients with suspected coronary artery disease and a normal electrocardiogram	Annals Of Internal Medicine	10.7326/0003-4819-147-12-200712180-00001
Le Gal 2006	Geneva score	Prediction of pulmonary embolism in the emergency department: the revised Geneva score	Annals of Internal Medicine	10.7326/0003-4819-144-3-200602070-00004
Levy 2006	Seattle Heart Failure Model	The Seattle Heart Failure Model prediction of survival in heart failure	Circulation	10.1161/CIRCULATIONAHA.105.584102
Pocock 2006	CHARM Risk Score	Predictors of mortality and morbidity in patients with chronic heart failure	European Heart Journal	10.1093/eurheartj/ehi555
Fonarow 2005	ADHERE Classification and Regression Tree (CART) Model	Risk stratification for in hospital mortality in acutely decompensated heart failure: classification and regression tree analysis	Journal of the American Medical Association	10.1001/jama.293.5.572
Kline 2004	PERC rule	Clinical criteria to prevent unnecessary diagnostic testing in emergency department patients with suspected pulmonary embolism	Journal of Thrombosis and Haemostasis	10.1111/j.1538-7836.2004.00790.x

Conroy 2003	SCORE	Estimation of ten-year risk of fatal cardiovascular disease in Europe: the SCORE project	European Heart Journal	10.1016/s0195-668x(03)00114-3
Lee 2003	EFFECT Risk Score	Predicting mortality among patients hospitalized for heart failure - Derivation and validation of a clinical model	Journal of the American Medical Association	10.1001/jama.290.19.2581
Assmann 2002	PROCAM score	Simple scoring scheme for calculating the risk of acute coronary events based on the 10-year follow-up of the prospective cardiovascular Münster (PROCAM) study	Circulation	10.1161/hc0302.102575
Gage 2001	CHADS2 score	Validation of clinical classification schemes for predicting stroke: results from the National Registry of Atrial Fibrillation	Journal of the American Medical Association	10.1001/jama.285.22.2864
Antman 2000	TIMI risk score	The TIMI risk score for unstable angina/non-ST elevation MI: A method for prognostication and therapeutic decision making	Journal of the American Medical Association	10.1001/jama.284.7.835
Well 2000	Wells score	Derivation of a simple clinical model to categorize patients probability of pulmonary embolism: increasing the models utility with the SimpliRED D-dimer	Thrombosis and Haemostasis	10.1055/s-0037-1613830
Kannel 1999	Framingham Heart Failure Risk Score	Profile for estimating risk of heart failure	Archives of Internal Medicine	10.1001/archinte.159.11.1197
Lee 1999	RCRI	Derivation and prospective validation of a simple index for prediction of cardiac risk of major noncardiac surgery	Circulation	10.1161/01.cir.100.10.1043
Aaronson 1997	Heart Failure Survival Score	Development and Prospective Validation of a Clinical Index to Predict Survival in Ambulatory Patients Referred for Cardiac Transplant Evaluation	Circulation	10.1161/01.cir.95.12.2660

Supplementary Table 9 Details of ROB Assessment in 20 Signaling Questions of PROBAST

No.	Signaling question	Yes/probably yes	No/probably no	No information
		n (%)	n (%)	n (%)
**Domain 1. Participants**				
1.1	Were appropriate data sources used, e.g., cohort, RCT, or nested case–control study data?	49 (86.0)	6 (10.5)	2 (3.5)
1.2	Were all inclusions and exclusions of participants appropriate?	54 (94.7)	1 (1.8)	2 (3.5)
**Domain 2. Predictors**				
2.1	Were predictors defined and assessed in a similar way for all participants?	55 (96.5)	0 (0)	2 (3.5)
2.2	Were predictor assessments made without knowledge of outcome data?	48 (84.2)	0 (0)	9 (15.8)
2.3	Are all predictors available at the time the model is intended to be used?	55 (96.5)	0 (0)	2 (3.5)
**Domain 3. Outcome**				
3.1	Was the outcome determined appropriately?	55 (96.5)	0 (0)	2 (3.5)
3.2	Was a prespecified or standard outcome definition used?	55 (96.5)	0 (0)	2 (3.5)
3.3	Were predictors excluded from the outcome definition?	53 (93.0)	2 (3.5)	2 (3.5)
3.4	Was the outcome defined and determined in a similar way for all participants?	55 (96.5)	0 (0)	2 (3.5)
3.5	Was the outcome determined without knowledge of predictor information?	48 (84.2)	0 (0)	9 (15.8)
3.6	Was the time interval between predictor assessment and outcome determination appropriate?	53 (93.0)	0 (0)	4 (7.0)
**Domain 4. Analysis**				
4.1	Were there a reasonable number of participants with the outcome?	54 (94.7)	1 (1.8)	2 (3.5)
4.2	Were continuous and categorical predictors handled appropriately?	52 (91.2)	3 (5.3)	2 (3.5)
4.3	Were all enrolled participants included in the analysis?	54 (94.7)	1 (1.8)	2 (3.5)
4.4	Were participants with missing data handled appropriately?	46 (80.7)	0 (0)	11 (19.3)
4.5	Was selection of predictors based on univariable analysis avoided?	46 (80.7)	6 (10.5)	5 (8.8)
4.6	Were complexities in the data (e.g., censoring, competing risks, sampling of control participants) accounted for appropriately?	54 (94.7)	0 (0)	3 (5.3)
4.7	Were relevant model performance measures evaluated appropriately?	31 (54.3)	23 (40.4)	3 (5.3)
4.8	Were model overfitting, underfitting, and optimism in model performance accounted for?	38 (66.7)	17 (29.8)	2 (3.5)
4.9	Do predictors and their assigned weights in the final model correspond to the results from the reported multivariable analysis?	49 (86.0)	0 (0)	8 (14.0)
